# Alterations in topology, cost, and dynamics of gamma-band EEG functional networks in a preclinical model of traumatic brain injury

**DOI:** 10.1162/netn.a.21

**Published:** 2025-07-29

**Authors:** Konstantinos Tsikonofilos, Michael Bruyns-Haylett, Hazel G. May, Cornelius K. Donat, Andriy S. Kozlov

**Affiliations:** Department of Bioengineering, Imperial College London, London, United Kingdom; Departments of Neuroscience and Clinical Neuroscience, Karolinska Institutet, Stockholm, Sweden; Departments of Bioengineering and Quantitative Methods, Institut Quimic de Sarria, Universitat Ramon Llul, Barcelona, Spain; Department of Brain Sciences, Imperial College London, London, United Kingdom; Helmholtz-Zentrum Dresden-Rossendorf, Institute of Radiopharmaceutical Cancer Research, Dresden, Germany

**Keywords:** TBI, EEG, Small-world, Rich-club, Cost, Epilepsy

## Abstract

Traumatic brain injury (TBI) is a major cause of disability leading to multiple sequelae in cognitive, sensory, and physical domains, including posttraumatic epilepsy. Despite extensive research, our understanding of its impact on macroscopic brain circuitry remains incomplete. We analyzed electrophysiological functional connectomes in the gamma band from an animal model of blast-induced TBI over multiple time points after injury. We revealed differences in small-world propensity and rich-club structure compared with age-matched controls, indicating functional reorganization following injury. We further investigated cost-efficiency trade-offs, propose a computationally efficient normalization procedure for quantifying the cost of spatially embedded networks that controls for connectivity strength differences, and observed dynamic changes across the injury timeline. To explore potential links between altered network topology and epileptic activity, we employed a brain-wide computational model of seizure dynamics and attribute brain reorganization to a homeostatic mechanism of activity regulation with the potential unintended consequence of driving generalized seizures. Finally, we demonstrated post-injury hyperexcitability that manifests as an increase in sound-evoked response amplitudes at the cortical level. Our work characterizes, for the first time, gamma-band functional network reorganization in a model of brain injury and proposes potential causes of these changes, thus identifying targets for future therapeutic interventions.

## Author Summary

Traumatic brain injury (TBI) is a prevalent neurological disorder caused by factors such as accidents, contact sports, or military conflict. While animal studies have provided detailed insights into the molecular and cellular mechanisms underlying TBI, and clinical research has revealed its effects on large-scale brain network organization, these findings often lack integration. Our research seeks to address this gap by examining large-scale brain activity in an animal model of TBI. By analyzing brain network structures, we identify reorganization, highlighting novel connections to weight gain and posttraumatic epilepsy. Further exploration using this model may deepen our understanding of cross-scale mechanisms and inform the development of therapeutic interventions.

## INTRODUCTION

Traumatic brain injury (TBI) affects millions annually (Maas et al., 2017) with post-injury phenotypes spanning sensory (Lew, Cifu, Crowder, & Grimes, 2012), physical (Golub & Reddy, 2022; Reid, Bragin, Giza, Staba, & Engel, 2016), cognitive (Adhikari et al., 2023; Silver, McAllister, & Arciniegas, 2009), and psychological (Silver et al., 2009) domains. Blast-induced TBI (bTBI) is prevalent in war zones (Hoge et al., 2008; Rona et al., 2012) and has been coined the signature injury of warfare (Martin, Lu, Helmick, French, & Warden, 2008), affecting both military personnel and civilians (Sattin, Sasser, Sullivent, & Coronado, 2008; Wade et al., 2008).

To date, a comprehensive understanding of how macroscopic TBI phenotypes emerge from the underlying pathophysiology remains elusive (Carron, Alwis, & Rajan, 2016). At the microscopic scale, Cantu et al. (2015) revealed signatures of an imbalance of excitation and inhibition (EI) as a precursor of posttraumatic epilepsy, a documented sequela of injury (Verellen & Cavazos, 2010). Indeed, many studies have highlighted the vulnerability of inhibitory neurons to TBI (Cantu et al., 2015; Frankowski, Kim, & Hunt, 2019; Hunt, Scheff, & Smith, 2011; Lowenstein, Thomas, Smith, & McIntosh, 1992; Nichols, Bjorklund, Newbern, & Anderson, 2018; Toth, Hollrigel, Gorcs, & Soltesz, 1997). Crucially, EI imbalance can lead to maladaptive plasticity as loss of inhibition and excessive firing can induce synaptic strengthening, and if uncontrolled, this process can lead circuits into a pathological regime (Chen, Epstein, & Stern, 2010). Recent studies have begun to map the multiscale reorganization of cortical circuits (Chen et al., 2010; Frankowski et al., 2022; Hunt et al., 2011; Vascak, Jin, Jacobs, & Povlishock, 2018), while Frankowski et al. (2022) identified a widespread restructuring of inhibitory networks following focal TBI.

Yet, another consequence of EI imbalance and excessive neuronal firing following TBI is metabolic stress. Restoring altered ionic gradients requires sodium-potassium pump activity, leading to elevated adenosine triphosphate (ATP) consumption, and this heightened ATP demand, coupled with mitochondrial dysfunction (Arun et al., 2013; Xiong, Gu, Peterson, Muizelaar, & Lee, 1997), can result in an energy crisis within neural tissue. This may disrupt the brain’s balance between energy cost and efficiency (Bullmore & Sporns, 2012), typically associated with “small-world” topology in brain networks. This topology combines densely interconnected clusters (low energetic costs) and short paths linking any two nodes (geodesic distance), ensuring efficient information transfer (Watts & Strogatz, 1998) as metabolic demands shape brain networks to balance efficient information transfer and minimal wiring cost (Bullmore & Sporns, 2012). Metabolic stress following TBI is expected to disrupt this balance, and studies have shown deviations from small-world topology in this context (Caeyenberghs et al., 2014; Cao & Slobounov, 2010; Pandit et al., 2013). However, an underexplored avenue involves conceptualizing functional brain networks as spatially embedded, considering physical rather than geodesic node distances and calculating network cost, assuming that strong or long-distance coupling incurs higher costs (Roy et al., 2017; van den Heuvel, Kahn, Goñi, & Sporns, 2012). This approach characterizes the cost-efficiency of network organization, aligning with theories on the emergence of small-world organization in biological networks (Bullmore & Sporns, 2012).

Another critical feature of brain networks is the presence of a highly interconnected core of hub nodes, often referred to as the “rich-club” structure, which supports computations and synchronization across the entire network (Kim & Min, 2020; van den Heuvel et al., 2012). Due to its strong coupling density, this functional backbone incurs elevated costs (van den Heuvel et al., 2012) and could also become disrupted in the context of a TBI-induced metabolic crisis. Crucially, insights from computational modeling suggest that this topology can influence the propensity of a network to generate seizures, while the contribution of individual nodes to this behavior is influenced by their centrality, that is, their hub-like characteristics (Lopes et al., 2017). These network measures might therefore also be disrupted following TBI, in the context of EI imbalance.

**Figure 1.**
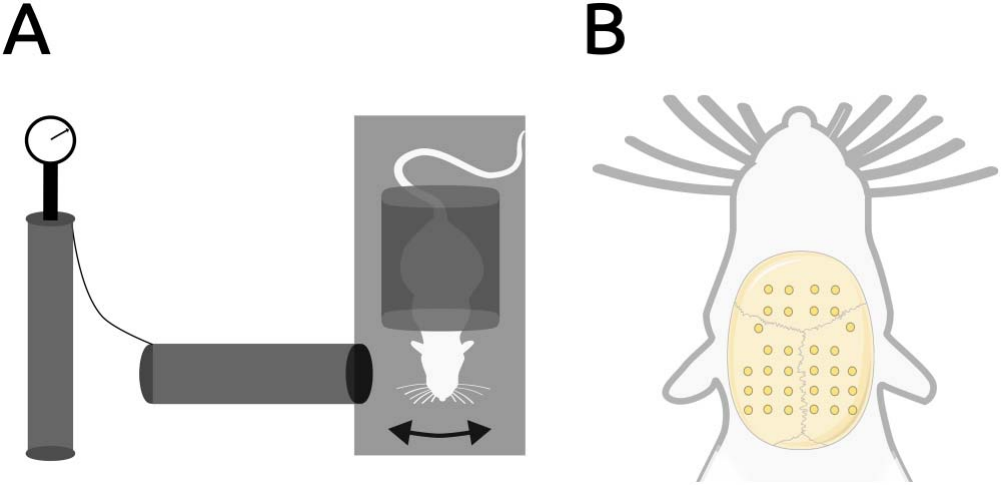
Top view schematic of the shock tube, including subject positioning, and of the electrode array. (A) The subject is positioned perpendicular to the incoming pressure shockwave, while the rest of the body besides the head is protected. The arrow illustrates that the subject’s head is allowed to move freely. The rest of the body besides the head is protected by a plastic tube with internal padding. (B) Macroscopic neural activity was recorded via epicranial EEG electrodes covering a large area of the skull. Adapted from Costa, G. (2020). Rat from the top. Zenodo. https://doi.org/10.5281/zenodo.3926343, licensed under CC BY 4.0.

**Figure 2.**
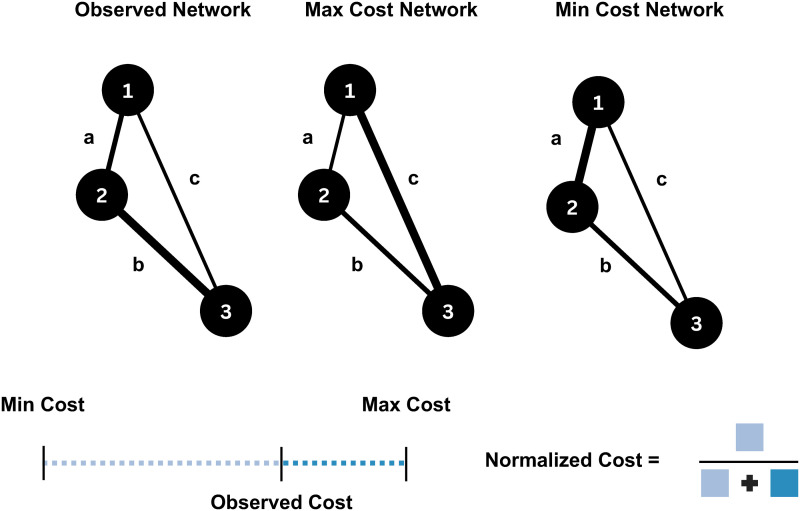
Illustration of our cost normalization procedure. For this example, we consider a simple, fully connected, three-node network with node names 1, 2, and 3. Furthermore, we consider this network to be spatially embedded, that is, nodes have physical distances from one another, that are of lengths a, b, and c, with *a* < *b* < *c*. We also assume an actual (observed) network where the three edges have weights reflected on edge width (leftmost network). We define the nonnormalized network cost as the sum of products of distance and weight over all network edges. Our normalization method pertains to finding permutations of the edge weights such that the permuted networks have the maximum or minimum cost attainable given the edge distances and weights (middle and rightmost networks). For this toy network, the maximum cost is achieved through a clockwise permutation of all edges (middle network), while the minimum cost is achieved by swapping edges {1,2} and {2,3}. In the general case, finding these global optima is performed analytically and, thus, comprises a computationally efficient operation. Finally, the normalized cost of the observed network is defined as the fractional deviation of the observed cost from the minimum cost, that is, the deviation from minimum cost divided by the range of attainable costs (bottom schematic). Thus, our normalized measure is bounded in the interval [0, 1].

Despite progress in TBI research, powerful analytical tools like network theory (Bassett & Sporns, 2017; Brynildsen, Rajan, Henderson, & Bassett, 2023; Farahani, Karwowski, & Lighthall, 2019; Heiney et al., 2021; Sharp, Scott, & Leech, 2014) and dynamic brain modeling (Pathak, Roy, & Banerjee, 2022; Sharp et al., 2014), along with translationally relevant recording modalities, have been underappreciated in preclinical models. In this work, we address this gap by further analyzing data from a previous study using a rat model of bTBI (May et al., 2024). We experimentally simulated blast pressure (Figure 1A) and recorded brain activity under anesthesia at 1 and 3 months post-injury using on-skull, multielectrode EEG (Figure 1B). Consistent with previous findings of hyperexcitability and altered connectivity post-injury, we observed elevated broadband spectral power and functional hyperconnectivity in the gamma frequency band (25–80 Hz), suggesting potential reorganization of distributed brain networks, associated with decreased inhibitory interneuron density. In the current work, we first analyze small-world propensity (SWP), a key topological property disrupted by TBI (Pandit et al., 2013), and hypothesize an energetic driver of this disruption reflected in network cost (Bullmore & Sporns, 2012) and reduction of costly topological features like rich-club structure. We introduce a novel metric for quantifying network cost, normalized to equivalent null models. SWP and rich-club structure were disrupted post-bTBI, while network cost and its efficiency trade-off showed a biphasic response: low cost at 1 month post-injury, high cost at 3 months, undetectable with the nonnormalized metric (Figure 2). This cost negatively correlated with weight gain, used as a proxy for the post-injury metabolic state. We also provide evidence for hyperexcitability in our model, reflected by increased auditory evoked potential amplitudes. Finally, we investigate the altered network topology’s impact on whole brain dynamics through computational modeling, observing increased seizure risk at the later timepoint, driven by most network nodes.

## RESULTS

### Blast-Induced Changes in the Topology of Resting Functional Networks

**Figure 3.**
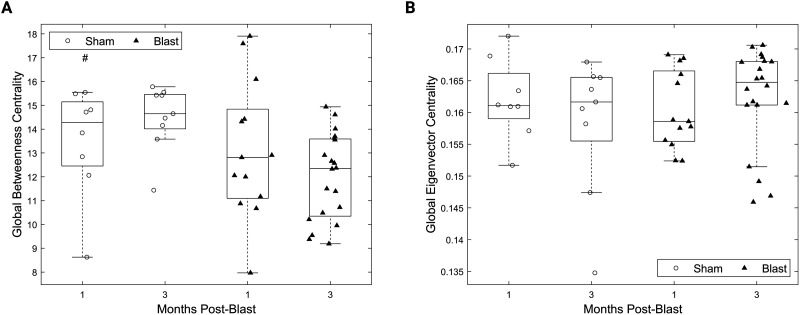
Decreased global centrality for the gamma connectivity networks chronically after injury. (A) Global betweenness centrality for all groups. (B) Global eigenvector centrality for all groups. All panels: Permutation-based two-way ANOVA (Group × Time Postblast with interaction, 10,000 permutations). Symbols denote *p* values for main effect of group. #: *p* < 0.05 (blast, 1 month: *n* = 13; blast, 3 months: *n* = 20; sham, 1 month: *n* = 8; sham, 3 months: *n* = 9).

Betweenness centrality (number of shortest paths in a network that traverse that node) was reduced at the global level after blast (Figure 3A; *F*(1, 46) = 4.89, *p* < 0.05, main effect of group). Eigenvector centrality (the centrality of a node as a function of the centrality of its neighbors) showed a trend towards increase for the injury group (Figure 3B; *p* > 0.05). Similarly, normalized betweenness centrality was not different between groups (Supporting Information Figure S1). No timepoint or interaction effects were detected for these measures.

**Figure 4.**
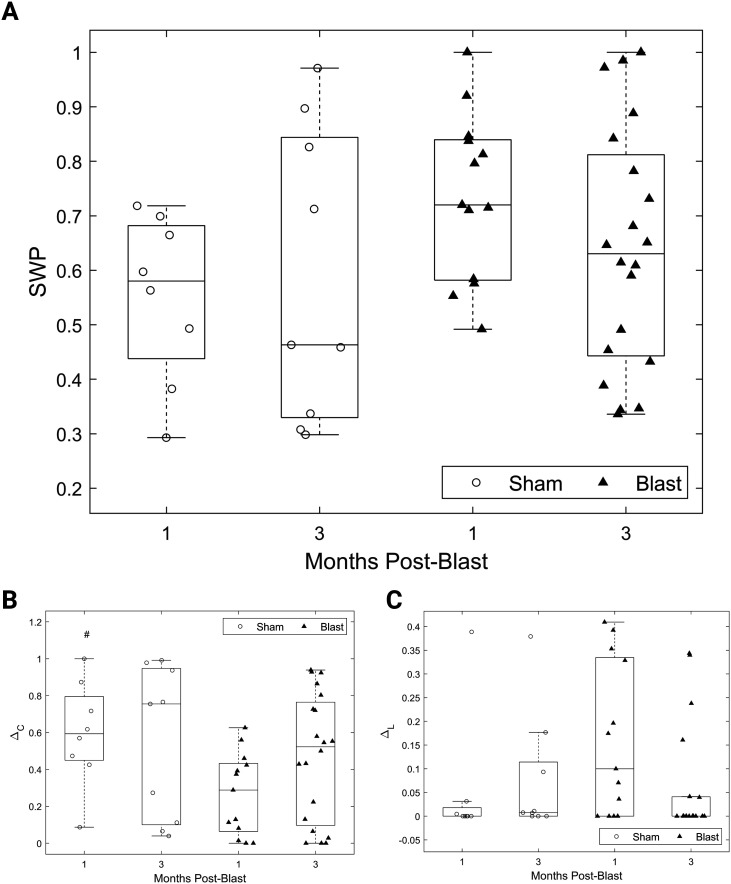
Decreased deviation from regular network clustering for the gamma connectivity networks after injury. (A) SWP for all groups. (B) Fractional deviation from the mean clustering coefficient of an equivalent regular network for all groups. (C) Fractional deviation from the mean path length of an equivalent random network for all groups. All panels: permutation-based two-way ANOVA (Group × Time Postblast with interaction, 10,000 permutations). Symbols denote *p* values for main effect of group. #: *p* < 0.05 (blast, 1 month: *n* = 13; blast, 3 months: *n* = 20; sham, 1 month: *n* = 8; sham, 3 months: *n* = 9).

In terms of whole-network measures, we observed a trend toward increased SWP in the blast group compared with the sham group (Figure 4A). The small-world topology combines the high clustering coefficients of regular and the short path lengths of random networks (Muldoon, Bridgeford, & Bassett, 2016; Watts & Strogatz, 1998). Hence, we dissected SWP into its constituents by analyzing the deviation of the mean clustering coefficient from that of an equivalent regular network and the deviation of the mean path length from that of an equivalent random network (Δ*_C_* and Δ_*L*, _ respectively; see the Materials and Methods section for more details) and revealed a reduced Δ*_C_* for the blast group at 1 month post-injury. This was accompanied by a larger deviation from the path length of an equivalent random network compared with the other experimental groups (Figure 4B–C). This trend was confirmed for Δ*_C_* (*F*(1, 46) = 4.20, *p* < 0.05, main effect of injury group) but did not reach significance for Δ*_L_* (*p* > 0.05). The presence of low values of Δ*_L_* across most of our cohort indicates that path lengths were identical to those of equivalent random networks. This result was robust to binarizing and thresholding networks across a wide range of thresholds (Supporting Information Figure S2). No timepoint or interaction effects were observed for these metrics.

**Figure 5.**
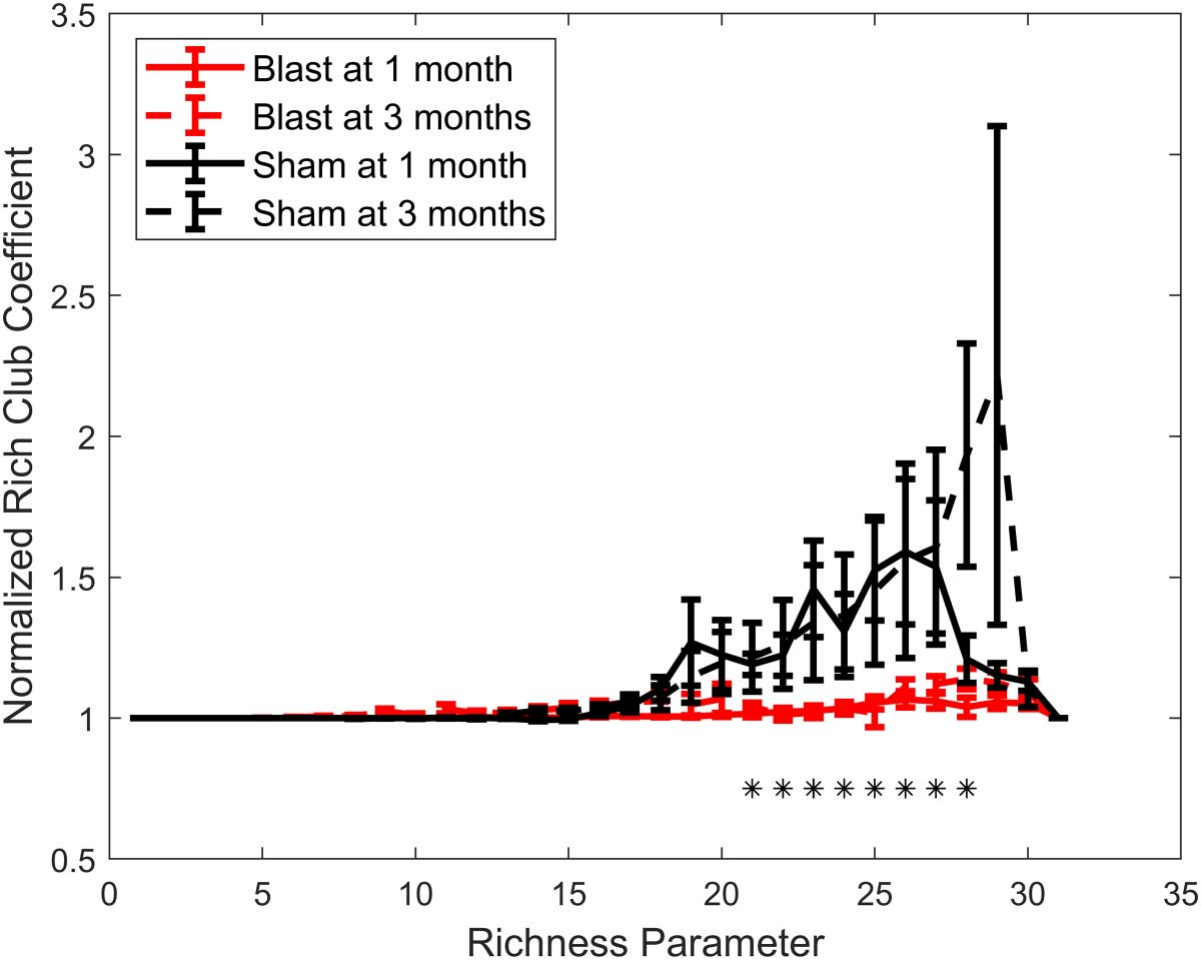
Loss of rich-club structure for the gamma connectivity networks chronically after injury. Permutation-based two-way ANOVA (Group × Time Post-Blast with interaction, 10,000 permutations) with FDR correction for multiple comparisons (*n* = 31 values of the richness parameter). Symbols denote corrected *p* values for main effect of group. *: *p* < 0.05 (blast, 1 month: *n* = 13; blast, 3 months: *n* = 20; sham, 1 month: *n* = 8; sham, 3 months: *n* = 9).

Finally, the presence of a rich-club structure was assessed using the normalized rich-club coefficient (Figure 5). Over a range of richness parameter values, a significant main effect of injury group was found with blast groups having diminished rich-club structure compared with sham groups.

### Blast-Induced Changes in Network Cost

The small-world topology is believed to achieve a cost-efficiency trade-off (Bullmore & Sporns, 2012), while the rich-club structure incurs high costs to a network (van den Heuvel et al., 2012). To identify putative drivers of the observed group differences in these features, we turn to network cost. Cost was first calculated similarly to Roy et al. (2017) as the inner product of euclidean distances and connectivity values across node pairs. However, this metric could be sensitive to group differences in connectivity. Hence, we propose a normalization procedure based on equivalent null models (see the Materials and Methods section). Briefly, we calculate lower and upper bounds of network cost for two inputs: a set of euclidean distances and a set of connectivity values. The normalized cost is then defined as the fractional deviation of the empirically observed cost from the lower cost bound. This measure is robust to differences in connectivity strength and can be interpreted as reflecting a “strategy” of resource (connectivity strengths) allocation to a system of prespecified properties (node distances), a potential plasticity-based phenomenon in the post-TBI brain. Our measure is computationally tractable, involving only a sorting operation of the distance and connectivity sets.

**Figure 6.**
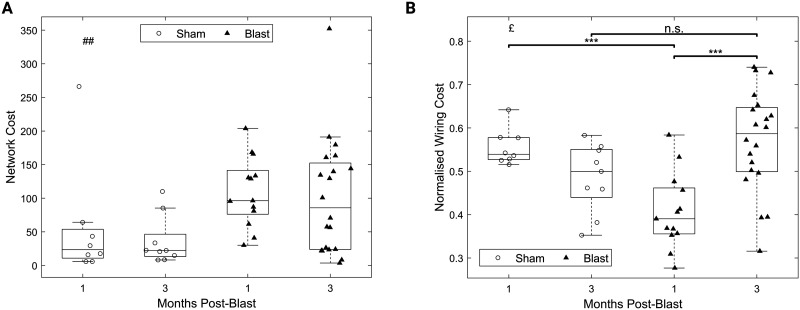
Dynamic changes in cost of gamma functional networks chronically after injury. (A) Nonnormalized network cost, similar to Roy et al. (2017). (B) Normalized network cost introduced here. All panels: permutation-based two-way ANOVA (Group × Time Postblast with interaction, 10,000 permutations). Symbols denote *p* values for main effect of group (#), interaction (£), or two-sample *t* test (*). £: *p* < 0.05; ##: *p* < 0.01; ***: *p* < 0.001 (blast, 1 month: *n* = 13; blast, 3 months: *n* = 20; sham, 1 month: *n* = 8; sham, 3 months: *n* = 9).

Network cost was elevated for the blast groups (*F*(1, 46) = 7.35, *p* < 0.01, main effect of group; Figure 6A) when using the nonnormalized cost metric. However, when considering our normalized cost measure, we observed a significant interaction effect (*F*(1, 46) = 16.85, *p* < 0.05; Figure 6B). Post hoc pairwise comparisons revealed a decreased normalized cost for the blast group at 1 month compared with the sham group at the same timepoint (*t*(30.37) = −4.60, *p* < 0.001, 95% CI [−0.23, −0.09]), and an elevated normalized cost for the blast group at 3 months compared with the blast group at 1 month (*t*(18.32) = −5.28, *p* < 0.001, 95% CI [−0.21, −0.09]). The blast group at 3 months was not found to be different to the sham group at 3 months.

**Figure 7.**
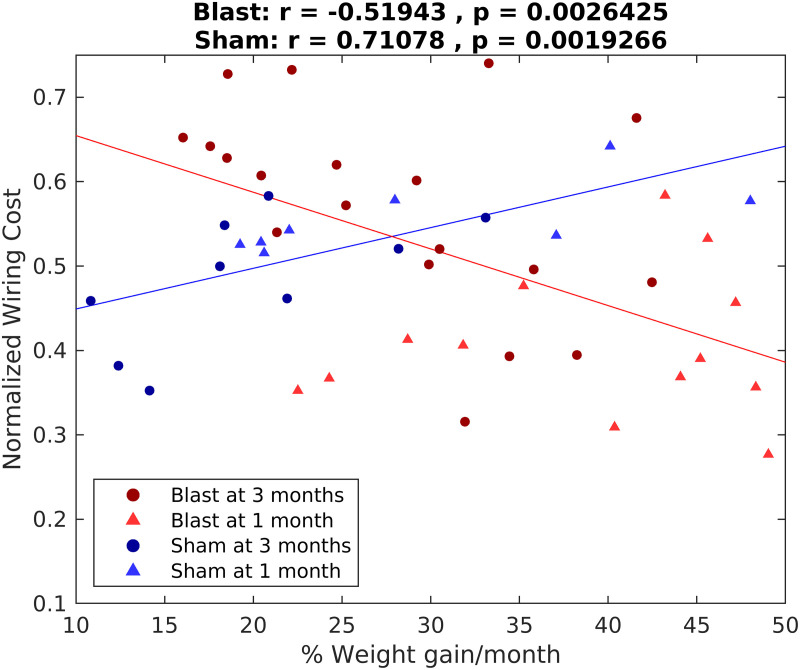
Opposite correlation signs between normalized network cost and normalized weight gain between sham and injury groups in the chronic phase. Spearman correlation values and associated *p* values are reported. Lines correspond to best fit lines using least squares (red line: blast groups; blue line: sham groups). Blast, 1 month: *n* = 13; blast, 3 months: *n* = 20; sham, 1 month: *n* = 8; sham, 3 months: *n* = 9.

Interestingly, our normalized cost metric was found to be significantly negatively correlated with normalized weight gain for the blast groups (Figure 7, red hue symbols; Spearman *ρ* = −0.52, 95% CI [−0.76, −0.17], *p* < 0.01), but positively correlated for the sham groups (Figure 7, blue hue symbols; Spearman *ρ* = 0.71, 95% CI [0.31,0.89], *p* < 0.01). This pattern was preserved when correlations were examined in all four subgroups independently except for the blast group measured at 1 month (Supporting Information Figure S3).

Alterations in network cost following TBI could reflect broader changes in cost-efficiency trade-offs (Bullmore & Sporns, 2012). Hence, we further integrate our cost measure in cost-efficiency metrics using the fractional deviations of SWP (see the Materials and Methods section) as deviations from efficiency. Specifically, we interpret clustering deviation from the equivalent regular network as segregative inefficiency and path length deviation from the equivalent random network as integrative inefficiency. We define high cost-efficiency as having low cost and efficiency deviations from their optimal values (see the Materials and Methods section). By virtue of their constituent quantities being normalized to appropriate null models, these measures are also robust to differences in overall density and connectivity strength.

**Figure 8.**
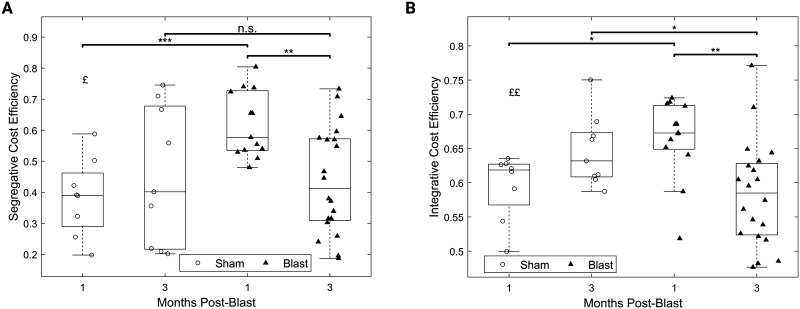
Dynamic changes in cost-efficiency of gamma functional networks chronically after injury. (A) Segragative cost-efficiency. (B) Integrative cost-efficiency. All panels: permutation-based two-way ANOVA (Group × Time Postblast with interaction, 10,000 permutations). Symbols denote *p* values for interaction effect (£) or two-sample *t* test (*). £, *: *p* < 0.05; ££, **: *p* < 0.01; ***: *p* < 0.001 (blast, 1 month: *n* = 13; blast, 3 months: *n* = 20; sham, 1 month: *n* = 8; sham, 3 months: *n* = 9).

An interaction effect was found for both segregative (Figure 8A; *F*(1, 46) = 6.04, *p* < 0.05) and integrative (Figure 8B; *F*(1, 46) = 11.86, *p* < 0.01) cost-efficiency, and post hoc pairwise comparisons revealed increased cost-efficiencies for the blast group at 1 month compared to the sham group at the same timepoint (segregative: *t*(13.07) = 4.13, *p* < 0.001, 95% CI [0.11, 0.34], Figure 8A; integrative: *t*(17.18) = 3.05, *p* < 0.05, 95% CI [0.02, 0.12], Figure 8B), as well as compared with the blast group at 3 months (segregative: *t*(31) = 3.69, *p* < 0.01, 95% CI [0.08, 0.28], Figure 8A; integrative: *t*(30.25) = 3.49, *p* < 0.01, 95% CI [0.03,0.13], Figure 8B). Decreased integrative cost-efficiency was found for the blast group at 3 months, compared with the sham group at the same timepoint (*t*(22.47) = −2.56, *p* < 0.05, 95% CI [−0.11, −0.01], Figure 8B).

### Blast-Induced Changes in Auditory Evoked Potentials

**Figure 9.**
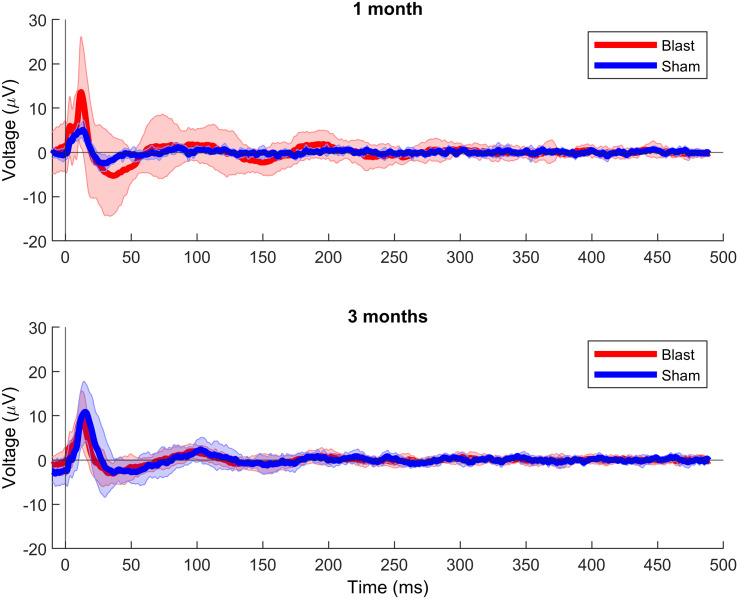
AEP waveforms for all groups—broadband clicks. Grand average waveforms for the 1-month (top) and 3-month groups (bottom). Shaded areas denote the standard deviation across the group. Blast, 1 month: *n* = 14; blast, 3 months: *n* = 20; sham, 1 month: *n* = 8; sham, 3 months: *n* = 10.

**Figure 10.**
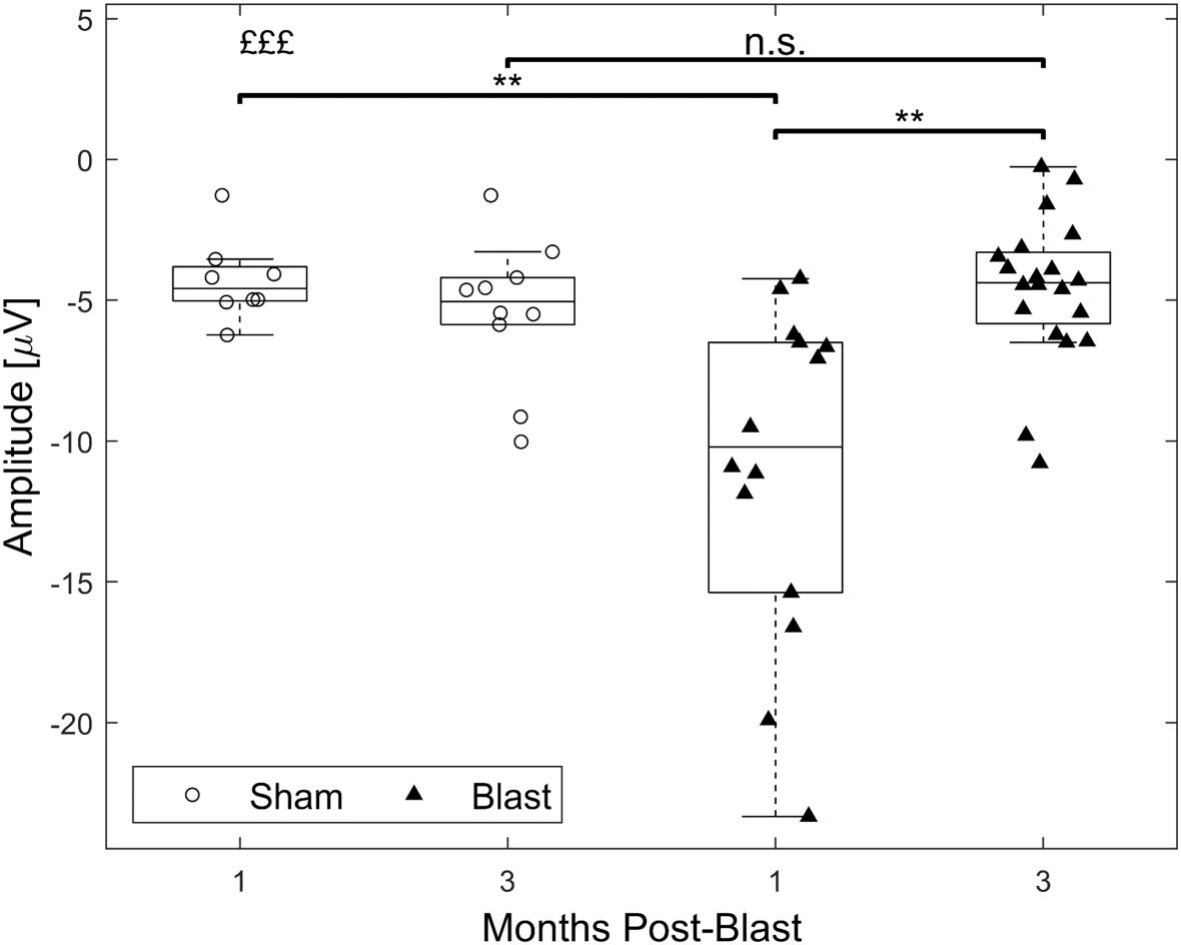
Increase in AEP N1 magnitudes 1 month after blast in response to broadband clicks. Permutation-based two-way ANOVA (Group × Time Postblast with interaction, 10,000 permutations). Symbols denote *p* values for an interaction effect (£) or two-sample *t* test (*). **: *p* < 0.01; £££: *p* < 0.001 (blast, 1 month: *n* = 14; blast, 3 months: *n* = 20; sham, 1 month: *n* = 8; sham, 3 months: *n* = 10).

As supporting evidence for the presence of a disrupted EI balance in our injury model, we probed the excitability of central auditory regions using evoked potentials. For broadband clicks, grand averages of evoked potential waveforms indicated increased amplitudes for the blast group, particularly at the 1 month timepoint (Figure 9). Analysis of the first negative component (N1) amplitude showed a significant interaction (*F*(1, 48) = 11.93, *p* < 0.001; Figure 10), with post hoc pairwise comparisons revealing a significant increase of magnitude for the blast group at 1 month compared with the sham group at the same timepoint (*t*(15.64) = −4.05, *p* < 0.01, 95% CI [−10.22, −3.19]) and to the blast group at 3 months (*t*(16.57) = −3.82, *p* < 0.01, 95% CI [−9.93, −2.85]). Similarly, increased amplitudes for the blast group were found for most other stimuli employed (Supporting Information Figures S6–S8).

### Blast-Induced Changes in Ictogenicity and the Influence of Topology

To decipher the role of altered network topology on seizure dynamics in the presence of hyperexcitability, we employed the brain ictogenicity framework (Lopes et al., 2017, 2019; Tait et al., 2021). Briefly, the functional connectivity values for each pair of nodes in sensor space are used as edge weights to define networks as for the graph-theoretical analysis above, while now, each node is imbued with dynamics that unfold over time. These dynamics are governed by a phase oscillator model operating in either a resting or a rotating dynamical regime, interpreted as resting or seizure dynamics, respectively (see the Materials and Methods section). Stronger coupling would result in higher ictogenicity and vice versa (e.g., see Figure 4a in Lopes et al., 2017). A control parameter *I*_0_ tunes the baseline excitability of individual nodes, on top of which, random noise and input currents from other nodes are summed to form the total current received by a node (Equation 12). This value is assigned to be equal across all nodes following previous work (Lopes et al., 2017; Tait et al., 2021). Subsequently, the network activity is simulated while parametrically varying this value across a range of negative values that do not drive the network into persistent epileptic dynamics (Tait et al., 2021). See the Connectivity-Informed Whole-Brain Computational Modeling section and Table 1 for more details on the simulation study. Outcome measures include seizure probability (*P*_*sz*_), brain network ictogenicity (BNI), and node ictogenicity (NI), the latter quantifying each node’s contribution to seizure probability in the whole network.

**Table 1.** Parameters of the computational model of brain ictogenicity

**Parameter**	**Meaning**	**Value**
*I*_0_	Node excitability	[−1.7, −0.5]
*K*	Global scaling of coupling strength	10
A	Connectivity matrix	dwPLI values scaled to [0, 1]
N	Network size	32
*σ*	Noise level	6
T	Simulation steps	4 ⋅ 10^6^
*δt*	Simulation time step	10^−2^

**Figure 11.**
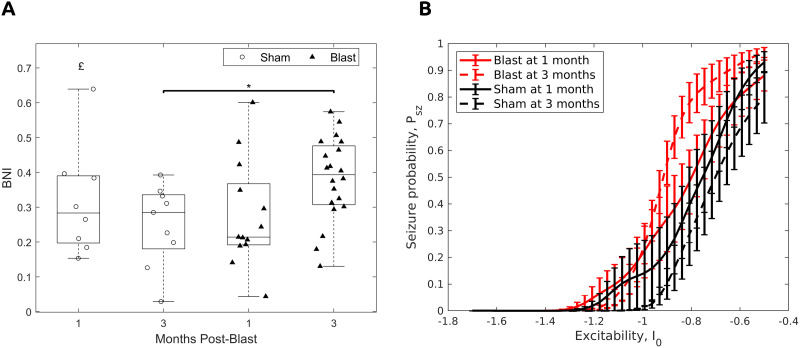
Increased network ictogenicity and seizure probability for the gamma functional networks 3 months post-injury. (A) BNI. Permutation-based two-way ANOVA (Group × Time Postblast with interaction, 10,000 permutations). Symbols denote *p* values for interaction effect (£) or two-sample *t* test (*). £, *: *p* < 0.05 (blast, 1 month: *n* = 13; blast, 3 months: *n* = 20; sham, 1 month: *n* = 8; sham, 3 months: *n* = 9). (B) Seizure probability (*P*_*sz*_) as a function of excitability (*I*_0_).

Globally, an interaction effect was found for BNI (Figure 11A; *F*(1, 46) = 4.47, *p* < 0.05). Post hoc pairwise comparisons revealed an increased BNI for the blast group at 3 months post-injury compared with the corresponding sham group (Figure 11A; *t*(15.84) = 2.78, *p* < 0.05, 95% CI [0.03, 0.23]). The plot of seizure probability (*P*_*sz*_) over excitability (*I*_0_) values revealed that the increased BNI for the blast group at 3 months was due to the increased seizure probability at intermediate to high excitability values (Figure 11B).

**Figure 12.**
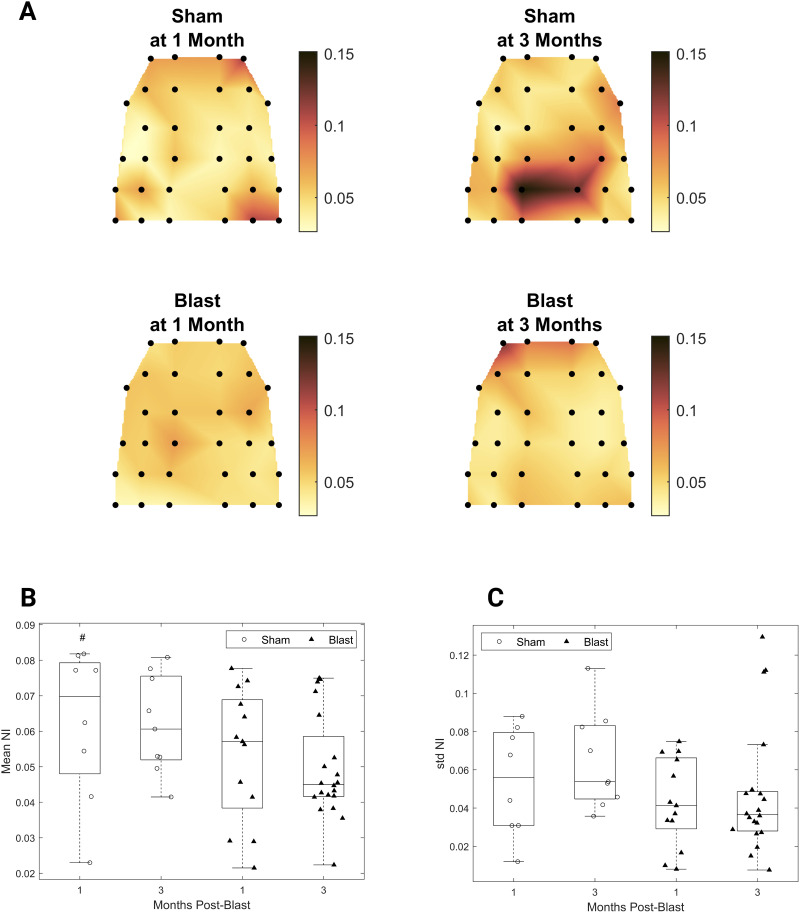
Decreased NI for the gamma-band functional networks chronically after injury. (A) Topoplots depicting mean group NI. (B) Mean NI. (C) Standard deviation of NI. (B, C) Permutation-based two-way ANOVA (Group × Time Postblast with interaction, 10,000 permutations). Symbols denote *p* values for the main effect of group. #: *p* < 0.05 (blast, 1 month: *n* = 13; blast, 3 months: *n* = 20; sham, 1 month: *n* = 8; sham, 3 months: *n* = 9).

NI quantifies the contribution of individual nodes to ictogenicity (Figure 12A). A significant effect of injury group was revealed for mean NI over nodes with decreased mean NI for the blast groups compared with the shams (Figure 12B; main effect of group, *F*(1, 46) = 4.40, *p* < 0.05). The standard deviation of NI over nodes showed a trend toward reduction for the blast groups across timepoints (Figure 12C).

As expected by the hub-like characteristics of a node as a proxy for its influence on network dynamics (Lopes et al., 2017), NI of individual nodes strongly correlated with centrality measures (Supporting Information Figure S4A, Spearman *ρ* = 0.6, 95% CI [0.57, 0.64], *p* < 0.001; Supporting Information Figure S4B, Spearman *ρ* = 0.85, 95% CI [0.83, 0.87], *p* < 0.001). Furthermore, mean NI correlated with the maximum value of the rich-club coefficient across richness parameter values (Supporting Information Figure S5; *β* = 0.02, 95% CI [0.015, 0.023], *t*(48) = 8.74, *p* < 0.001, adjusted *R*^2^ = 0.611).

## DISCUSSION

In this study, we observed changes in gamma-band functional network topology post-injury. Locally, betweenness centrality decreased. Globally, networks showed increased SWP due to higher clustering, and a pronounced loss of rich-club structure. Network costs decreased at 1 month post-injury, recovering by the third month, a pattern only discernible with our normalized metric. Network cost correlated with post-injury weight gain, positively in sham and negatively in blast cohorts. We found evidence of post-injury hyperexcitability through increased auditory evoked potential amplitudes. Computational modeling revealed increased network ictogenicity at 3 months post-injury and a reduced role of individual nodes in epileptogenesis, significantly correlated with centrality and rich-club structure.

Our findings align with prior research. Altered small-worldness has been reported in EEG-based TBI studies (Cao & Slobounov, 2010), and MRI-based studies have observed increased path lengths (Caeyenberghs et al., 2014; Pandit et al., 2013). Changes within the gamma frequency range suggests a plastic reorganization of inhibitory networks (Hunt et al., 2011; Vascak et al., 2018), as these neurons shape activity in this band (Cardin et al., 2009) and are impacted by injury in our model (May et al., 2024). Supporting this, Frankowski et al. (2022) documented increased local and reduced long-range input to inhibitory interneurons, even in distant areas from the injury locus. We observed this effect along with a disruption in the rich-club structure, consistent with a diffusion imaging study (Verhelst, Vander Linden, De Pauw, Vingerhoets, & Caeyenberghs, 2018). Magnetoencephalography (MEG) studies have shown changes in rich-club hub representation (Antonakakis, Dimitriadis, Zervakis, Papanicolaou, & Zouridakis, 2017a) and hyperactivation of rich-club networks (Antonakakis, Dimitriadis, Zervakis, Papanicolaou, & Zouridakis, 2017b), echoing fMRI observations (Hillary et al., 2014; Li et al., 2022).

To identify drivers of altered small-worldness, we investigated the cost-efficiency trade-off thought to be afforded by the small-world topology. This trade-off is usually quantified using cost metrics incorporating connection distance and strength (Roy et al., 2017; van den Heuvel et al., 2012). A recent modeling study (Achterberg, Akarca, Strouse, Duncan, & Astle, 2023) suggests that artificial neural networks optimizing performance and cost converge to a small-world architecture. However, topological measures should be compared with appropriate null models, as confounding factors such as weight distribution may spuriously drive group differences in topological measures (Váša & Mišić, 2022), an important confound in TBI-induced connectivity strength differences. We integrated these arguments by introducing a novel, computationally efficient normalization procedure for calculating cost in weighted networks based on equivalent null models. This revealed a complex post-TBI network cost trajectory, indiscernible with the standard metric. The trajectory of diminished cost in early chronic stages followed by an increase in later stages might suggest a temporally evolving metabolic perturbation post-injury. Literature connects TBI with such aberrations (Bergsneider et al., 2000; Kawamata, Katayama, Hovda, Yoshino, & Becker, 1992; McGill et al., 2022; Shiga et al., 2006; Sullivan, Keller, Mattson, & Scheff, 1998; Vallez Garcia, Otte, Dierckx, & Doorduin, 2016; Xiong et al., 1997), noting a multiphasic response typified by initial hypermetabolism followed by hypometabolism and potential recovery (Koenig & Dulla, 2018). Alternatively, the increased cost for the blast group at 3 months relative to the blast group at 1 month might reflect a shift toward more efficient information processing by allocating high-weight edges to distant node pairs. This could reflect a transition away from a low-cost, low-efficiency topology that does not satisfy the behavioral needs of the organism. Importantly, post-TBI cerebral glucose metabolism measured by positron emission tomography (PET) has been positively correlated with full-scale intelligence quotient scores (Kato et al., 2007), corroborating the existence of trade-offs between energy conversion and cognition.

Interestingly, there was a reversal in the correlation direction between our proposed network cost metric and weight gain across groups. Our metric, interpreted as a proxy for resource allocation strategy (lower cost reflecting a lower part of the cost range given network distances and connectivity strengths), suggests potential differences in energy intake due to TBI. In a typical brain, increased energy supply might allow elevated network costs, promoting enhanced computational capabilities. In contrast, putative TBI-induced metabolic challenges could drive increased food intake and employ a more frugal resource allocation strategy, reducing network costs. This would make weight gain inversely associated with network cost, as shown here. The restored costs at 3 months post-injury could reflect a compensatory response. Specifically, the reduced cost at 1 month might curtail computational capabilities enough to disrupt normal function, necessitating a shift to a more resource-intensive configuration to offset these challenges. These findings on cost dynamics align with cost-efficiency analyses, supporting this interpretation.

The localization of these results in the gamma frequency band could also provide converging evidence for a metabolic driver in the observed effects, as fast-spiking inhibitory interneurons’ activity is energetically demanding (Kann, Papageorgiou, & Draguhn, 2014). Kann (2016) hypothesized that these neural subtypes might be preferentially affected by TBI through an energetic insult. Our present and previous (May et al., 2024) findings support this hypothesis and extend it to the macroscopic spatial scale and reorganization of brain-wide networks as measured by EEG electrodes.

The rich-club structure is postulated to function as a densely interconnected core that integrates information processed by peripheral components (Kim & Min, 2020; van den Heuvel et al., 2012). Hence, this functional backbone incurs a high cost (van den Heuvel et al., 2012). Consequently, a metabolic perspective could also explain the observed post-injury disruption of this topological feature observed in this study. However, a complementary perspective from our computational modeling emphasizes neural dynamics. The rich-club’s capacity to facilitate activity propagation can be a double-edged sword. While beneficial for information transfer in a healthy brain, this could, in the backdrop of TBI and its associated hyperexcitability, induce uncontrolled activation cascades, possibly culminating in seizures. Lopes et al. (2017) demonstrated that targeted elimination of rich-club nodes curtails network ictogenicity in silico, and this intervention corresponded with favorable outcomes in postresective surgery of epileptic patients. Thus, the loss of rich-club organization post-TBI might signify an adaptive strategy to counteract excessive activity spread and mitigate seizure risks, especially amid EI imbalances. Importantly, our interpretation rests on the assumption that changes in edge-level features such as the topological properties investigated here occur in response to node-level changes such as disrupted EI balance, in order to mitigate their effects on the rest of the network. Our findings indicate an opposing effect of injury on global and local ictogenicity with interesting implications. On one hand, network-wide seizure probability remains unchanged at 1 month but is increased by the third month post-injury, compared with shams at the same timepoint. In parallel, the mean contribution of each node as well as the heterogeneity in these contributions decreases for the blast group, suggesting that all nodes equally drive the network toward this more prominent epileptic regime. This could reflect the interplay of local and global topological changes both between injury groups and across the injury timeline that are shared (like rich-club organization) or unique (small-worldness or cost). Finally, the switch-like behavior observed at the later timepoint, combined with the decreased contribution of individual nodes to seizures throughout the whole network, could suggest a potential substrate for generalized seizures rather than focal seizures, as observed in a study using data from clinical populations with these two types of epilepsy (Lopes et al., 2019). The reduction in individual node contribution, as well as its correlation with topological measures, lends support to the interpretation of an adaptive loss of rich-club structure. Intriguingly, a similar pattern in global ictogenicity and node-level contributions has been identified in research focusing on Alzheimer’s disease (Tait et al., 2021), suggesting a potentially general adaptive mechanism applicable across diverse neurological conditions.

The influence of injury on evoked potentials was prominent, pointing to hyperexcitability (Bruyns-Haylett et al., 2017) in cortical auditory regions and echoing increased amplitudes in pediatric epileptic patients (Zgorzalewicz, 2006). This pattern, combined with disruptions to inhibitory interneurons across multiple brain regions in the same model (May et al., 2024), reinforces our interpretation of the observed network differences as indicative of a disrupted EI balance (Carron et al., 2016). Although we cannot rule out a hearing loss–related driver of this phenotype (Popelar, Grecova, Rybalko, & Syka, 2008; Rumschlag & Razak, 2021), recent research in a Fragile X syndrome model (Jonak, Lovelace, Ethell, Razak, & Binder, 2020) noted a similar trend of increased auditory evoked potential amplitudes. Given that this syndrome shares a mechanistic substrate of hyperexcitability, it is likely that a significant portion of the effects observed here stem from bTBI. Future studies could further explore the interplay between network topology and node excitability on ictogenicity by incorporating heterogeneous excitability across network nodes, for example, by using a proxy measure such as the power spectral exponent (Gao, Peterson, & Voytek, 2017).

Overall, the graph theoretical characterization of functional networks has yielded a nuanced view of injury consequences. Supplemented by a new methodological contribution in calculating network cost, which shows promise of increased sensitivity to experimental effects while controlling for potential confounds, this approach has integrated electrophysiology with a metabolic proxy post-TBI. Computational modeling results suggest that these alterations might be the brain’s response as part of a homeostatic mechanism to maintain dynamics post-injury. The study also reiterates the dynamic nature of injury progression and the potential role of the inhibitory plasticitome (McFarlan et al., 2023), aligning with the hypothesis that TBI reopens the early developmental critical period where the EI balance has not yet stabilized, and plastic changes occur more frequently and intensely (Masri, 2020).

Recording brain activity during an anesthetized state is a major limitation of the current work, and future investigations should record activity in awake behaving rodents (Choi, Koch, Poppendieck, Lee, & Shin, 2010; Jonak, Lovelace, Ethell, Razak, & Binder, 2018; Jonak et al., 2020). We do, however, expect some of our results to transfer to the awake state, based on evidence on the robustness of gamma-band connectivity differences to anesthetic state when using urethane (Mondino et al., 2024) as well as the similarity of fMRI-based functional connectivity patterns to those observed during wakefulness (Paasonen, Stenroos, Salo, Kiviniemi, & Gröhn, 2018). Importantly, unlike other anesthetics that target specific receptors (e.g., isoflurane that targets GABAergic receptors (Jones, Brooks, & Harrison, 1992) and influences gamma-band oscillations (Hudetz, Vizuete, & Pillay, 2011)), urethane has a nonspecific mechanism of action (Hara & Harris, 2002), making it an appropriate choice for studies investigating EI balance. However, this anesthetic has been shown to modulate the excitability (Schumacher, Schneider, & Woolley, 2011), geometry of population activity (Kobak, Pardo-Vazquez, Valente, Machens, & Renart, 2019), and spike rate and representational capacity (Pachitariu, Lyamzin, Sahani, & Lesica, 2015) but not the activity structure (Harris et al., 2011) of auditory neurons or inhibitory gating in nonauditory regions (Wang, Ma, Wang, & Qin, 2018). Incorporating the collection of behavioral data into future study designs would also help the speculated functional significance of the electrophysiological changes observed in the current study to be elucidated. Moreover, the suspected metabolic disruptions in this model and their association with the proposed network cost metric should be validated by future studies using additional techniques able to directly interrogate brain metabolism such as PET scanning. Finally, using a longitudinal within-subjects design could improve sensitivity to experimental effects that dynamically unfold over the post-injury period. By benchmarking our cost metric using larger datasets from clinical cohorts, its applicability can be gauged against other energetically relevant neural measures (Chennu et al., 2017; Smailovic et al., 2020).

## MATERIALS AND METHODS

### Experimental Procedures

All animal experiments were conducted in compliance with the Home Office License ([Scientific Procedures] Act 1986 and European Union legislation). Briefly, male Sprague Dawley rats were used, housed in standard conditions. We employed a 2 × 2 between-subjects factorial design based on injury group (sham or blast) and post-injury timepoint (1 or 3 months). Blast exposure was conducted using a shock tube (Campos-Pires et al., 2023; Eftaxiopoulou et al., 2016; Kazezian, Yu, Ramette, Macdonald, & Bull, 2021; T.-T. Nguyen, 2016; T.-T. Nguyen et al., 2019; T.-T. N. Nguyen, Sory, Amin, Rankin, & Proud, 2018; T.-T. N. Nguyen, Wilgeroth, & Proud, 2014), and the injury was unilaterally delivered on the right side of isoflurane-anesthetized animals, isolated to the head, which was allowed to move freely (Figure 1A). Animals in the blast group received a pressure wave with peak overpressure of 232.5826(6.4486) kPa and positive phase duration of 1.3870(0.0427) ms (both values *M*(*SD*)), while the sham animals received an identical procedure minus the pressure wave. Electrophysiological recordings involved terminal anesthesia under urethane, and 32-channel flexible EEG multielectrode arrays and a SmartBox Pro/Radiens Allego system (all electrophysiological equipment by Neuronexus, Michigan, USA). Electrodes were implanted on the dorsal skull surface and span 11.7 mm on the rostrocaudal and 9.6 mm on the left-to-right axis, with a minimum interelectrode distance of 1.2 mm (Figure 1B; Neuronexus, Rat EEG Functional). Signals were referenced to the nuchal musculature and sampled at 30 kHz. More details can be found in the Supporting Information and in our previous work (May et al., 2024).

### Analysis of Functional Connectomes

#### Data preprocessing and connectome construction.

Data preprocessing was performed using functions from the EEGLAB Toolbox (Delorme & Makeig, 2004) and custom scripts in MATLAB (Version 2019b). Briefly, signals were downsampled to 1 kHz and line noise was removed using the *CleanLine* plugin (Bigdely-Shamlo, Mullen, Kothe, Su, & Robbins, 2015; Mullen, 2012). This was followed by automatic artifact rejection (EEGLAB function *clean_artifacts*; less than three electrodes were rejected per recording and all accepted ones were used for further analysis) and an independent-component decomposition using the Infomax algorithm (function *pop_runica*) to separate artifactual components. Components visually inspected those containing primarily respiratory-, cardiac-, or muscle-related artifacts were conservatively removed from the data. Three animals were excluded due to noisy data. More details on preprocessing can be found in the Supporting Information.

Functional connectivity was assessed using the debiased weighted phase-lag index (dwPLI; Vinck, Oostenveld, van Wingerden, Battaglia, & Pennartz, 2011), using MATLAB and FieldTrip functions. dwPLI is a frequency-resolved, phase-based metric quantifying the consistency of phase differences between two time series, interpreted as the time delay for neural activity to reach the target region (Cohen, 2014). Importantly, phase differences are weighted by magnitude such that zero-phase connectivity (which might be attributable to volume-conduction in sensor-level analysis) is discarded (Vinck et al., 2011). Signals were segmented into 3-s windows and the cross-spectral density for each electrode pair was calculated using a 1.5-s sliding window and 50% overlap (MATLAB function *cpsd*). dwPLI values were calculated using the FieldTrip function *ft_connectivity_wpli*. Connectivity values for a frequency band are determined by taking the mean across the frequency bins specific to the gamma band (25–80 Hz).

#### Local topological measures.

Topological analysis of connectomes was conducted using custom scripts, MATLAB functions, functions from the Brain Connectivity Toolbox (BCT; Rubinov, Kötter, Hagmann, & Sporns, 2009), and publicly available code (Muldoon et al., 2016).

To characterize local structure, we quantified the importance of network nodes via centrality measures. *Betweenness centrality* (Matlab function *centrality*) is defined as the number of shortest paths in the network that transverse that node, while *eigenvector centrality* (BCT function *eigenvector_centrality_und*) measures the centrality (importance) of a node as a function of the centrality of its neighbors (Lohmann et al., 2010).

Global variants of node-specific measures were calculated as the mean across the network. Since betweenness centrality can be influenced by additive shifts of the weight distribution, a normalized measure was also computed by dividing the global centrality of the observed networks by the mean global centrality of 100 equivalent random networks.

#### Global topological measures.

To characterize global topology, we employed SWP (Muldoon et al., 2016), which quantifies the small-world characteristics of a network by positioning it along the continuum from regular to random (as in the original formulation from Watts & Strogatz, 1998) using appropriate null models. This measure quantifies for the observed network, the deviation of its clustering coefficient Δ*_C_*, from an equivalent regular network, and the deviation of its path length Δ*_L_*, from an equivalent random network and is computed according to the following equation:

$$SWP=1-\sqrt{\frac{\Delta_{C}^{2}+\Delta_{L}^{2}}{2}}$$ (1)

The deviations are calculated as follows:

$$\Delta_{C}=\frac{C_{reg}-C_{obs}}{C_{reg}-C_{\mathrm{rand}}}$$ (2)

$$\Delta_{L}=\frac{L_{obs}-L_{\mathrm{rand}}}{L_{reg}-L_{\mathrm{rand}}}$$ (3)

where *obs, reg, rand* refer to the observed, equivalent regular, and equivalent random networks, respectively.

To calculate the clustering coefficients, the measure by Onnela, Saramäki, Kertész, and Kaski (2005) is used:

$$C_{i}=\frac{1}{k_{i}\left(k_{i}-1\right)}\sum_{j,k}{\left({\hat{w}}_{ij}{\hat{w}}_{jk}{\hat{w}}_{ik}\right)}^{1/3}$$ (4)

where *C*_*i*_ is the clustering coefficient of node *i, k*_*i*_ the number of edges adjacent to node *i*, and ${\hat{w}}_{ij}$ the weight of the edge between nodes *i* and *j* divided by the maximum network weight.

For path lengths, edge weights is defined as the functional coupling strength between adjacent nodes; hence, nodes connected by a strong edge can be interpreted as being in close proximity from a neural communication perspective. Hence, we interpret inverse edge weight as distance (Muldoon et al., 2016). Shortest paths are calculated using Dijkstra’s algorithm and is used to characterize the whole network:

$$L=\frac{1}{N\left(N-1\right)}\sum_{i\neq j}d_{ij}$$ (5)

where *N* is the number of nodes and *d*_*ij*_ the length of the shortest path connecting nodes *i* and *j*.

To generate equivalent random networks, observed network edges are randomly reassigned. For equivalent regular networks, edges are organized in descending order and are distributed along the subdiagonals of the adjacency matrix. These equivalent networks preserve the weight distribution, making them appropriate in the context of TBI.

Rich-club structure was assessed using the approach by van den Heuvel et al. (2013). Specifically, the rich-club coefficient was calculated for each value of the richness parameter defined as the node degree (BCT function *rich_club_wu*), and the same calculation was performed for 100 equivalent random networks (BCT function *randmio_und_connected*, which ensures that the randomized network maintains connectedness). Intuitively, the rich-club structure can be understood as the presence of strong coupling between the hub (i.e., rich in connections) nodes of a network. Finally, the *normalized* rich-club coefficient was calculated as the ratio of the observed coefficient to the mean of the coefficients of the random networks, for each value of the richness parameter.

#### A novel normalization procedure for cost quantification.

The concept of network cost has been particularly illuminating in the context of TBI. A notable study (Roy et al., 2017) determined network cost by computing the dot product of the functional connectivity matrix and the physical distance matrix, based on the premise that connections that are strongly coupled or spatially distant demand higher energetic expenditure.

In the context of TBI, as with the network topology measures previously discussed, variations in cost—given a network’s spatial configuration (physical distances between nodes)—might trivially arise simply due to disparities in overall connectivity strength. Consequently, to draw valid comparisons between networks, it is necessary to employ a suitable normalization method or a null model. To this end, we introduce the concept of total effective wiring cost (TEWC). This metric is derived by initially determining the cost of the observed network, following the methodology of Roy et al. (2017), where the cost is the dot product of edge weights and corresponding Euclidean distances between the adjacent nodes:

$$C=\sum_{i\neq j}d_{i,j}w_{i,j}$$ (6)

where *d*_*i, j*_ denoted the node distances and *w*_*i, j*_ the corresponding edge weights.

Following this, two surrogate null networks are constructed via reassignment of edge weights, representing the extremes of the cost spectrum for the given configuration of the network: the *minimum cost equivalent network* and the *maximum cost equivalent network*. Constructed to incur either the minimal or maximal cost, these networks maintain the sets of physical distances and connection strengths between nodes, and their costs can be calculated using Equation 6. Subsequently, the TEWC is defined as the fractional deviation from the minimum cost, relative to the maximum cost attainable:

$$\text{TEWC}=\frac{C_{obs}-C_{\min}}{C_{\max}-C_{\min}}$$ (7)

where the subscripts *obs, min, max* refer to the observed, maximum cost equivalent, and minimum cost equivalent networks, respectively.

This formulation can be interpreted in terms of a strategy of resource allocation (i.e., weights) across a set of nodes with fixed physical distances. Thus, it is suitable to quantify potential network reorganization schemes that may be implemented after injury by plasticity mechanisms, for example, as an adaptive response to a post-TBI energy crisis (Bergsneider et al., 2000; Giza & Hovda, 2014; Kawamata et al., 1992; McGuire et al., 2019; Shiga et al., 2006; Sullivan et al., 1998; Vallez Garcia et al., 2016; Xiong et al., 1997).

This reduction of the problem to one of edge allocation naturally leads to an efficient implementation through a closed-form solution, making use of the rearrangement inequality (Hardy, Littlewood, & Pólya, 1952). Given two equally sized sets of real numbers *x*_1_, *x*_2_, . . , *x*_*n*_ and *y*_1_, *y*_2_, …, *y*_*n*_ sorted in ascending order, the following inequality holds:

$$x_{n}y_{1}+x_{n-1}y_{2}+\ldots +x_{1}y_{n}\leq x_{\sigma \left(1\right)}y_{1}+x_{\sigma \left(2\right)}y_{2}+\ldots +x_{\sigma \left(n\right)}y_{n}\leq x_{1}y_{1}+x_{2}y_{2}+\ldots +x_{n}y_{n}$$ (8)

for every permutation *σ* of the indices *1, 2, …, n*.

It follows that the cost calculation for the equivalent maximum and minimum cost networks described above reduces to sorting the weights of the observed network in descending and ascending order and taking their dot product with the sorted distance vector according to ascending order, yielding the cost of the minimum- and maximum-cost equivalent networks, respectively. Having a closed form, this metric is computationally efficient and reduces to one sorting operation per network. Our implementation makes use of the *sort* function of the core MATLAB package, which, in turn, employs an implementation of the Quick Sort algorithm (Hoare, 1961). This algorithm has an average performance of $O\left(n\log n\right)$, pointing to the scalability of TEWC for parcellations involving a large number of brain regions.

Brain organization does not strictly minimize cost (Bullmore & Sporns, 2012), instead striking a balance between efficient organization and information processing capacity. Consequently, cost metrics are often evaluated within the framework of cost-efficiency (Bassett et al., 2009), quantifying this aforementioned equilibrium.

Here, we introduce two metrics of cost-efficiency by combining the newly introduced TEWC with the Δ*_C_* and Δ*_L_* deviations, yielding segregative and integrative cost-efficiency (SCE and ICE, respectively) as described below:

$$SCE=1-\sqrt{\frac{\Delta_{C}^{2}+{\text{TEWC}}^{2}}{2}}$$ (9)

$$ICE=1-\sqrt{\frac{\Delta_{L}^{2}+{\text{TEWC}}^{2}}{2}}$$ (10)

Analogously to how SWP gauges the coexistence of short path lengths with pronounced clustering, these cost-efficiency metrics evaluate the presence of low cost alongside computational capacity. Here, higher computational capacity is quantified as smaller deviation from the null equivalent models encompassed by the SWP framework, namely, the equivalent regular and random networks. This, in turn, allows for a systematic decomposition of cost-efficiency into components associated with segregation and integration.

### Evoked Potentials Analysis

Auditory stimuli were 0.1-ms-long broadband clicks, as well as 25-ms-long tone pips at 10, 37.5, and 65 kHz, all at an intensity of 80 dB SPL. Stimuli were repeated 1,000 times with an interstimulus interval of 500 ms using ultrasonic dynamic speakers (Vifa, Avisoft). Electrophysiological data under auditory stimulation were downsampled to 2 kHz, and line noise was removed as described above. Voltage excursions exceeding 400 *μ*V were also removed and data filtered between 3 and 300 Hz using the EEGLAB function *pop_eegfiltnew*. Finally, data were segmented into epochs and meaned over trials to extract evoked potential waveforms. More details on stimuli and data preprocessing can be found in the Supporting Information.

For the analysis of auditory evoked potentials, the approach and terminology used by Jonak et al. (2020) are adopted. For each electrode, the N1 component was automatically identified using the MATLAB function *findpeaks* within a predetermined time window (15 to 100 ms poststimulus). Subsequently, the peak amplitude was extracted and stored for group-level analysis.

### Connectivity-Informed Whole-Brain Computational Modeling

#### Simulations using a phase-oscillator model.

Networks of nodes were defined based on the functional connectomes, with nodes corresponding to electrodes (32 nodes for a full dataset) and edge weights set based on functional connectivity (dwPLI) values. Node dynamics were simulated using the following theta model (Ermentrout & Kopell, 1986):

$${\dot{\theta }}_{j}=\left(1-\cos\theta_{j}\right)+\left(1+\cos\theta_{j}\right)I_{j}\left(t\right)$$ (11)

$$I_{j}\left(t\right)=I_{0}^{\left(j\right)}+{\sigma \xi }^{\left(j\right)}\left(t\right)+\frac{K}{N}\sum_{i\neq j}\alpha_{ij}[1-\cos(\theta_{i}-\theta_{i}^{\left(s\right)}]$$ (12)

$$\theta_{i}^{\left(s\right)}=-ℜ\cos^{-1}\left(\frac{1+I_{0}^{\left(i\right)}}{1-I_{0}^{\left(i\right)}}\right)$$ (13)

where *θ_j_* is the phase of node *j, I*_*j*_(*t*) is the input current received by node *j* at time *t*, $I_{0}^{\left(j\right)}$ is the excitability of node *j, ξ*^(*j*)^(*t*) are noisy inputs to node *j* from distant regions, *α_ij_* is the coupling strength between nodes *i* and *j, K* is a global scaling constant, *N* is the number of nodes, and $\theta_{i}^{\left(s\right)}$ is the stable phase of the oscillators in the absence of coupling and noise at which the values of *θ* were initialized for all nodes. Simulations were run for a total of 6 million timesteps and repeated five times, while the values of the excitability parameter *I*_0_ were varied.

Values for *K* and *I*_0_ were selected similarly to Tait et al. (2021). Specifically, *I*_0_ was varied from −1.7 to −0.5 in 40 equally spaced values across simulations for a functional connectome. Connectivity matrices were scaled to [0,1] by division with the maximum value in a static fashion, that is, based on the whole recording and were kept fixed for each simulation for that subject while *I*_0_ is varied. The specific parameter values used are detailed in Table 1.

Code accompanying the publication by Tait et al. (2021) available from GitHub (https://github.com/lukewtait/AlzheimersBNI.git) was used for simulations.

#### Outcome measures of ictogenicity.

Following the simulations, two summary measures of the dynamics were calculated. Globally, BNI is defined as the integral of the seizure probability over the excitability parameter values (Lopes et al., 2017):

$$BNI=\int_{\lambda_{1}}^{\lambda_{2}}P_{sz}\left(\lambda \right)d\lambda$$ (14)

where the seizure probability is defined as the proportion of simulation time during which the system is engaged in epileptic dynamics:

$$P_{sz}\left(I_{0}\right)=\frac{1}{N}\sum_{i=1}^{N}\frac{t_{sz}^{\left(i\right)}\left(I_{0}\right)}{T}$$ (15)

At the node level, NI quantifies the contribution of each node to the whole network’s BNI:

$${NI}^{\left(i\right)}=\frac{{BNI}_{pre}-{BNI}_{\text{post}}^{\left(i\right)}}{{BNI}_{pre}}$$ (16)

where *BNI*_*pre*_ and *BNI*_*post*_ are the BNI of the full network and the BNI following the removal of node *i*, respectively. Importantly, NI stands as a normalized metric, ensuring that potential group effects are not spuriously driven by differences in global connectivity strength.

### Statistical Analysis

Permutation-based two-way analysis of variance (ANOVA) tests (Anderson, 2001; Anderson & Braak, 2003) were conducted, including terms for the main effect of group, main effect of timepoint, and an interaction effect. This procedure is robust to deviations from normality and outlier values but is not unaffected by violations of homoscedasticity. It can be argued, however, that variance changes in the outcome measures of an experimental group (particularly the injury group) is indeed an effect of interest and sensitivity to it is desirable.

In the case of a significant interaction effect, post hoc pairwise comparisons were performed for the contrasts of interest (blast at 1 month vs. blast at 3 months, blast at 1 month vs. sham at 1 month, blast at 3 months vs. sham at 3 months), using permutation-based two-sample *t* tests (MATLAB function *ttest2* with unequal variances from the statistics and machine learning toolbox), from which the confidence interval for the difference of population means was also extracted.

Correlations between continuous variables were assessed using Spearman correlation (MATLAB function *corr*). Ninety-five percent confidence intervals were calculated by bootstrapping (10,000 samples). In the presence of outlier observations, a robust regression approach was followed (MATLAB function *fitlm* with default parameters).

For tests examining group pairs for post hoc pairwise analyses, correction for multiple comparisons was performed using the Bonferroni correction method (VanderWeele & Mathur, 2019). When considering values within a parameterized space (such as the richness or excitability parameters), the Benjamini-Hochberg false discovery rate method was used (Benjamini & Hochberg, 1995). The significance threshold was set at 0.05 for adjusted *p* values in all tests.

In all figures, “#” denotes a main effect of injury group, while “%” denotes an interaction effect. “*” denotes a significant effect in two-sample *t* tests. The number of symbols has the usual interpretation (one symbol: *p* < 0.05, two symbols: *p* < 0.01, three symbols: *p* < 0.001).

## Acknowledgments

We acknowledge Gil Costa for the use of the scientific figure from SciDraw (DOI: doi.org/10.5281/zenodo.3926343), which was adapted for this work. We also thank SciDraw for providing open-access scientific illustrations that enhance scientific communication. Additionally, we acknowledge Servier Medical Art (licensed under CC BY 4.0) for their medical illustrations.

## Author Contributions

Konstantinos Tsikonofilos: Conceptualization; Data curation; Formal analysis; Investigation; Methodology; Software; Visualization; Writing – original draft; Writing – review & editing. Michael Bruyns-Haylett: Conceptualization; Data curation; Investigation; Methodology; Project administration; Supervision; Writing – review & editing. Hazel G. May: Investigation; Writing – review & editing. Cornelius K. Donat: Investigation; Writing – review & editing. Andriy S. Kozlov: Conceptualization; Funding acquisition; Methodology; Project administration; Supervision; Writing – review & editing.

## Funding Information

Andriy Kozlov, Royal British Legion (https://dx.doi.org/10.13039/100008631). Andriy Kozlov, Army Research Office (https://dx.doi.org/10.13039/100000183).

## Supplementary Material



## TECHNICAL TERMS

Blast-induced traumatic brain injury: Brain injury resulting from exposure to explosions, causing damage to the brain’s structure and function.
Balance of excitation and inhibition: Equilibrium between nerve cell activation and suppression, crucial for maintaining stable neural activity and proper brain function.
Epilepsy: A neurological disorder causing recurrent seizures, characterized by abnormal electrical activity in the brain.
Hyperexcitability: A state of increased neuronal activity or responsiveness in the nervous system.
Auditory evoked potentials: Brain responses triggered by auditory stimuli, usually measured through electrodes on the scalp, providing insights into auditory processing.
Critical period: Sensitive developmental phase during which specific experiences or stimuli have a profound and lasting impact on an organism’s development.
Shock tube: A device generating controlled shock waves for research, testing materials, or simulating blast effects, often used in scientific experiments.
Inhibitory interneurons: Neurons that dampen the activity of other neurons, playing a key role in controling the overall excitability of neural networks.
Homeostatic mechanism: Regulatory process maintaining internal stability by adjusting physiological variables to counteract changes, promoting overall equilibrium in the system.

## References

[bib1] Achterberg, J., Akarca, D., Strouse, D. J., Duncan, J., & Astle, D. E. (2023). Spatially embedded recurrent neural networks reveal widespread links between structural and functional neuroscience findings. Nature Machine Intelligence, 5, 1369–1381. 10.1038/s42256-023-00748-9

[bib2] Adhikari, A., Brooks, J., Watson, K., Morris, E. E., LoBue, C., Motes, M., … Chiang, H.-S. (2023). A-133 self-reported loss of consciousness predicts executive functions in veterans with a history of traumatic brain injury. *Archives of Clinical Neuropsychology*, 38(7), 1305. 10.1093/arclin/acad067.150

[bib3] Anderson, M., & Braak, C. T. (2003). Permutation tests for multi-factorial analysis of variance. Journal of Statistical Computation and Simulation, 73(2), 85–113. 10.1080/00949650215733

[bib4] Anderson, M. J. (2001). Permutation tests for univariate or multivariate analysis of variance and regression. Canadian Journal of Fisheries and Aquatic Sciences, 58(3), 626–639. 10.1139/f01-004

[bib5] Antonakakis, M., Dimitriadis, S. I., Zervakis, M., Papanicolaou, A. C., & Zouridakis, G. (2017a). Altered rich-club and frequency-dependent subnetwork organization in mild traumatic brain injury: A MEG resting-state study. Frontiers in Human Neuroscience, 11, 416. 10.3389/fnhum.2017.00416, 28912698 PMC5582079

[bib6] Antonakakis, M., Dimitriadis, S. I., Zervakis, M., Papanicolaou, A. C., & Zouridakis, G. (2017b). Reconfiguration of dominant coupling modes in mild traumatic brain injury mediated by δ-band activity: A resting state MEG study. Neuroscience, 356, 275–286. 10.1016/j.neuroscience.2017.05.032, 28576727

[bib7] Arun, P., Abu-Taleb, R., Oguntayo, S., Wang, Y., Valiyaveettil, M., Long, J. B., & Nambiar, M. P. (2013). Acute mitochondrial dysfunction after blast exposure: Potential role of mitochondrial glutamate oxaloacetate transaminase. Journal of Neurotrauma, 30(19), 1645–1651. 10.1089/neu.2012.2834, 23600763

[bib8] Bassett, D. S., Bullmore, E. T., Meyer-Lindenberg, A., Apud, J. A., Weinberger, D. R., & Coppola, R. (2009). Cognitive fitness of cost-efficient brain functional networks. Proceedings of the National Academy of Sciences, 106(28), 11747–11752. 10.1073/pnas.0903641106, 19564605 PMC2703669

[bib9] Bassett, D. S., & Sporns, O. (2017). Network neuroscience. Nature Neuroscience, 20(3), 353–364. 10.1038/nn.4502, 28230844 PMC5485642

[bib10] Benjamini, Y., & Hochberg, Y. (1995). Controlling the false discovery rate: A practical and powerful approach to multiple testing. Journal of the Royal Statistical Society: Series B (Methodological), 57(1), 289–300. 10.1111/j.2517-6161.1995.tb02031.x

[bib11] Bergsneider, M., Hovda, D. A., Lee, S. M., Kelly, D. F., McArthur, D. L., Vespa, P. M., … Becker, D. P. (2000). Dissociation of cerebral glucose metabolism and level of consciousness during the period of metabolic depression following human traumatic brain injury. Journal of Neurotrauma, 17(5), 389–401. 10.1089/neu.2000.17.389, 10833058

[bib12] Bigdely-Shamlo, N., Mullen, T., Kothe, C., Su, K.-M., & Robbins, K. A. (2015). The PREP pipeline: Standardized preprocessing for large-scale EEG analysis. Frontiers in Neuroinformatics, 9, 16. 10.3389/fninf.2015.00016, 26150785 PMC4471356

[bib13] Bruyns-Haylett, M., Luo, J., Kennerley, A. J., Harris, S., Boorman, L., Milne, E., … Zheng, Y. (2017). The neurogenesis of P1 and N1: A concurrent EEG/LFP study. NeuroImage, 146, 575–588. 10.1016/j.neuroimage.2016.09.034, 27646129 PMC5312787

[bib14] Brynildsen, J. K., Rajan, K., Henderson, M. X., & Bassett, D. S. (2023). Network models to enhance the translational impact of cross-species studies. Nature Reviews Neuroscience, 24(9), 575–588. 10.1038/s41583-023-00720-x, 37524935 PMC10634203

[bib15] Bullmore, E., & Sporns, O. (2012). The economy of brain network organization. Nature Reviews Neuroscience, 13(5), 336–349. 10.1038/nrn3214, 22498897

[bib16] Caeyenberghs, K., Leemans, A., Leunissen, I., Gooijers, J., Michiels, K., Sunaert, S., & Swinnen, S. P. (2014). Altered structural networks and executive deficits in traumatic brain injury patients. Brain Structure and Function, 219(1), 193–209. 10.1007/s00429-012-0494-2, 23232826

[bib17] Campos-Pires, R., Ong, B. E., Koziakova, M., Ujvari, E., Fuller, I., Boyles, C., … Dickinson, R. (2023). Repetitive, but not single, mild blast TBI causes persistent neurological impairments and selective cortical neuronal loss in rats. Brain Sciences, 13(9), 1298. 10.3390/brainsci13091298, 37759899 PMC10526452

[bib18] Cantu, D., Walker, K., Andresen, L., Taylor-Weiner, A., Hampton, D., Tesco, G., & Dulla, C. G. (2015). Traumatic brain injury increases cortical glutamate network activity by compromising gabaergic control. Cerebral Cortex, 25(8), 2306–2320. 10.1093/cercor/bhu041, 24610117 PMC4494035

[bib19] Cao, C., & Slobounov, S. (2010). Alteration of cortical functional connectivity as a result of traumatic brain injury revealed by graph theory, ICA, and sLORETA analyses of EEG signals. IEEE Transactions on Neural Systems and Rehabilitation Engineering, 18(1), 11–19. 10.1109/TNSRE.2009.2027704, 20064767 PMC2945220

[bib20] Cardin, J. A., Carlén, M., Meletis, K., Knoblich, U., Zhang, F., Deisseroth, K., … Moore, C. I. (2009). Driving fast-spiking cells induces gamma rhythm and controls sensory responses. Nature, 459(7247), 663–667. 10.1038/nature08002, 19396156 PMC3655711

[bib21] Carron, S. F., Alwis, D. S., & Rajan, R. (2016). Traumatic brain injury and neuronal functionality changes in sensory cortex. Frontiers in Systems Neuroscience, 10, 47. 10.3389/fnsys.2016.00047, 27313514 PMC4889613

[bib22] Chen, H., Epstein, J., & Stern, E. (2010). Neural plasticity after acquired brain injury: Evidence from functional neuroimaging. PM & R, 2, S306–S312. 10.1016/j.pmrj.2010.10.006, 21172692

[bib23] Chennu, S., Annen, J., Wannez, S., Thibaut, A., Chatelle, C., Cassol, H., … Laureys, S. (2017). Brain networks predict metabolism, diagnosis and prognosis at the bedside in disorders of consciousness. Brain, 140(8), 2120–2132. 10.1093/brain/awx163, 28666351

[bib24] Choi, J. H., Koch, K. P., Poppendieck, W., Lee, M., & Shin, H.-S. (2010). High resolution electroencephalography in freely moving mice. Journal of Neurophysiology, 104(3), 1825–1834. 10.1152/jn.00188.2010, 20610789

[bib25] Cohen, M. X. (2014). *Analyzing neural time series data: Theory and practice*. MIT Press. 10.7551/mitpress/9609.001.0001

[bib26] Delorme, A., & Makeig, S. (2004). EEGLAB: An open source toolbox for analysis of single-trial EEG dynamics including independent component analysis. Journal of Neuroscience Methods, 134(1), 9–21. 10.1016/j.jneumeth.2003.10.009, 15102499

[bib27] Eftaxiopoulou, T., Barnett-Vanes, A., Arora, H., Macdonald, W., Nguyen, T.-T. N., Itadani, M., … Rankin, S. M. (2016). Prolonged but not short-duration blast waves elicit acute inflammation in a rodent model of primary blast limb trauma. Injury, 47(3), 625–632. 10.1016/j.injury.2016.01.017, 26838938

[bib28] Ermentrout, G. B., & Kopell, N. (1986). Parabolic bursting in an excitable system coupled with a slow oscillation. SIAM Journal on Applied Mathematics, 46(2), 233–253. 10.1137/0146017

[bib29] Farahani, F. V., Karwowski, W., & Lighthall, N. R. (2019). Application of graph theory for identifying connectivity patterns in human brain networks: A systematic review. Frontiers in Neuroscience, 13, 585. 10.3389/fnins.2019.00585, 31249501 PMC6582769

[bib30] Frankowski, J. C., Kim, Y. J., & Hunt, R. F. (2019). Selective vulnerability of hippocampal interneurons to graded traumatic brain injury. Neurobiology of Disease, 129, 208–216. 10.1016/j.nbd.2018.07.022, 30031783 PMC6690377

[bib31] Frankowski, J. C., Tierno, A., Pavani, S., Cao, Q., Lyon, D. C., & Hunt, R. F. (2022). Brain-wide reconstruction of inhibitory circuits after traumatic brain injury. Nature Communications, 13(1), 3417. 10.1038/s41467-022-31072-2, 35701434 PMC9197933

[bib32] Gao, R., Peterson, E. J., & Voytek, B. (2017). Inferring synaptic excitation/inhibition balance from field potentials. NeuroImage, 158, 70–78. 10.1016/j.neuroimage.2017.06.078, 28676297

[bib33] Giza, C. C., & Hovda, D. A. (2014). The new neurometabolic cascade of concussion. Neurosurgery, 75(0 4), S24–S33. 10.1227/NEU.0000000000000505, 25232881 PMC4479139

[bib34] Golub, V. M., & Reddy, D. S. (2022). Post-traumatic epilepsy and comorbidities: Advanced models, molecular mechanisms, biomarkers, and novel therapeutic interventions. Pharmacological Reviews, 74(2), 387–438. 10.1124/pharmrev.121.000375, 35302046 PMC8973512

[bib35] Hara, K., & Harris, R. A. (2002). The anesthetic mechanism of urethane: The effects on neurotransmitter-gated ion channels. Anesthesia & Analgesia, 94(2), 313–318. 10.1213/00000539-200202000-00015, 11812690

[bib36] Hardy, G. H., Littlewood, J. E., & Pólya, G. (1952). *Inequalities*. Cambridge University Press.

[bib37] Harris, K. D., Bartho, P., Chadderton, P., Curto, C., de la Rocha, J., Hollender, L., … Sakata, S. (2011). How do neurons work together? Lessons from auditory cortex. Hearing Research, 271(1–2), 37–53. 10.1016/j.heares.2010.06.006, 20603208 PMC2992581

[bib38] Heiney, K., Huse Ramstad, O., Fiskum, V., Christiansen, N., Sandvig, A., Nichele, S., & Sandvig, I. (2021). Criticality, connectivity, and neural disorder: A multifaceted approach to neural computation. Frontiers in Computational Neuroscience, 15, 611183. 10.3389/fncom.2021.611183, 33643017 PMC7902700

[bib39] Hillary, F. G., Rajtmajer, S. M., Roman, C. A., Medaglia, J. D., Slocomb-Dluzen, J. E., Calhoun, V. D., … Wylie, G. R. (2014). The rich get richer: Brain injury elicits hyperconnectivity in core subnetworks. PLoS One, 9(8), e104021. 10.1371/journal.pone.0104021, 25121760 PMC4133194

[bib40] Hoare, C. A. R. (1961). Algorithm 64: quicksort. Communications of the ACM, 4(7), 321. 10.1145/366622.366644

[bib41] Hoge, C. W., McGurk, D., Thomas, J. L., Cox, A. L., Engel, C. C., & Castro, C. A. (2008). Mild traumatic brain injury in US soldiers returning from Iraq. New England Journal of Medicine, 358(5), 453–463. 10.1056/NEJMoa072972, 18234750

[bib42] Hudetz, A. G., Vizuete, J. A., & Pillay, S. (2011). Differential effects of isoflurane on high-frequency and low-frequency γ oscillations in the cerebral cortex and hippocampus in freely moving rats. Anesthesiology, 114(3), 588–595. 10.1097/ALN.0b013e31820ad3f9, 21293253 PMC3123712

[bib43] Hunt, R. F., Scheff, S. W., & Smith, B. N. (2011). Synaptic reorganization of inhibitory hilar interneuron circuitry after traumatic brain injury in mice. Journal of Neuroscience, 31(18), 6880–6890. 10.1523/JNEUROSCI.0032-11.2011, 21543618 PMC3102318

[bib44] Jonak, C. R., Lovelace, J. W., Ethell, I. M., Razak, K. A., & Binder, D. K. (2018). Reusable multielectrode array technique for electroencephalography in awake freely moving mice. Frontiers in Integrative Neuroscience, 12, 53. 10.3389/fnint.2018.00053, 30416434 PMC6213968

[bib45] Jonak, C. R., Lovelace, J. W., Ethell, I. M., Razak, K. A., & Binder, D. K. (2020). Multielectrode array analysis of EEG biomarkers in a mouse model of Fragile X Syndrome. Neurobiology of Disease, 138, 104794. 10.1016/j.nbd.2020.104794, 32036032 PMC9038039

[bib46] Jones, M. V., Brooks, P. A., & Harrison, N. L. (1992). Enhancement of gamma-aminobutyric acid-activated Cl-currents in cultured rat hippocampal neurones by three volatile anaesthetics. Journal of Physiology, 449(1), 279–293. 10.1113/jphysiol.1992.sp019086, 1326046 PMC1176079

[bib47] Kann, O. (2016). The interneuron energy hypothesis: Implications for brain disease. Neurobiology of Disease, 90, 75–85. 10.1016/j.nbd.2015.08.005, 26284893

[bib48] Kann, O., Papageorgiou, I. E., & Draguhn, A. (2014). Highly energized inhibitory interneurons are a central element for information processing in cortical networks. Journal of Cerebral Blood Flow & Metabolism, 34(8), 1270–1282. 10.1038/jcbfm.2014.104, 24896567 PMC4126088

[bib49] Kato, T., Nakayama, N., Yasokawa, Y., Okumura, A., Shinoda, J., & Iwama, T. (2007). Statistical image analysis of cerebral glucose metabolism in patients with cognitive impairment following diffuse traumatic brain injury. Journal of Neurotrauma, 24(6), 919–926. 10.1089/neu.2006.0203, 17600509

[bib50] Kawamata, T., Katayama, Y., Hovda, D. A., Yoshino, A., & Becker, D. P. (1992). Administration of excitatory amino acid antagonists via microdialysis attenuates the increase in glucose utilization seen following concussive brain injury. Journal of Cerebral Blood Flow & Metabolism, 12(1), 12–24. 10.1038/jcbfm.1992.3, 1345756

[bib51] Kazezian, Z., Yu, X., Ramette, M., Macdonald, W., & Bull, A. M. J. (2021). Development of a rodent high-energy blast injury model for investigating conditions associated with traumatic amputations. Bone & Joint Research, 10(3), 166–173. 10.1302/2046-3758.103.BJR-2020-0367.R1, 33663228 PMC7998070

[bib52] Kim, D.-J., & Min, B.-K. (2020). Rich-club in the brain’s macrostructure: Insights from graph theoretical analysis. Computational and Structural Biotechnology Journal, 18, 1761–1773. 10.1016/j.csbj.2020.06.039, 32695269 PMC7355726

[bib53] Kobak, D., Pardo-Vazquez, J. L., Valente, M., Machens, C. K., & Renart, A. (2019). State-dependent geometry of population activity in rat auditory cortex. eLife, 8, e44526. 10.7554/eLife.44526, 30969167 PMC6491041

[bib54] Koenig, J. B., & Dulla, C. G. (2018). Dysregulated glucose metabolism as a therapeutic target to reduce post-traumatic epilepsy. Frontiers in Cellular Neuroscience, 12, 350. 10.3389/fncel.2018.00350, 30459556 PMC6232824

[bib55] Lew, H. L., Cifu, D. X., Crowder, A. T., & Grimes, J. B. (2012). Sensory and communication disorders in traumatic brain injury. Journal of Rehabilitation Research and Development, 49(7), VII–X. 10.1682/JRRD.2012.07.0126, 23341287

[bib56] Li, F., Liu, Y., Lu, L., Shang, S., Chen, H., Haidari, N. A., … Chen, Y.-C. (2022). Rich-club reorganization of functional brain networks in acute mild traumatic brain injury with cognitive impairment. Quantitative Imaging in Medicine and Surgery, 12(7), 3932–3946. 10.21037/qims-21-915, 35782237 PMC9246720

[bib57] Lohmann, G., Margulies, D. S., Horstmann, A., Pleger, B., Lepsien, J., Goldhahn, D., … Turner, R. (2010). Eigenvector centrality mapping for analyzing connectivity patterns in fmri data of the human brain. PLoS One, 5(4), e10232. 10.1371/journal.pone.0010232, 20436911 PMC2860504

[bib58] Lopes, M. A., Perani, S., Yaakub, S. N., Richardson, M. P., Goodfellow, M., & Terry, J. R. (2019). Revealing epilepsy type using a computational analysis of interictal EEG. Scientific Reports, 9(1), 10169. 10.1038/s41598-019-46633-7, 31308412 PMC6629665

[bib59] Lopes, M. A., Richardson, M. P., Abela, E., Rummel, C., Schindler, K., Goodfellow, M., & Terry, J. R. (2017). An optimal strategy for epilepsy surgery: Disruption of the rich-club? PLoS Computational Biology, 13(8), e1005637. 10.1371/journal.pcbi.1005637, 28817568 PMC5560820

[bib60] Lowenstein, D. H., Thomas, M. J., Smith, D. H., & McIntosh, T. K. (1992). Selective vulnerability of dentate hilar neurons following traumatic brain injury: A potential mechanistic link between head trauma and disorders of the hippocampus. Journal of Neuroscience, 12(12), 4846–4853. 10.1523/JNEUROSCI.12-12-04846.1992, 1464770 PMC6575779

[bib61] Maas, A. I. R., Menon, D. K., Adelson, P. D., Andelic, N., Bell, M. J., Belli, A., … InTBIR Participants and Investigators. (2017). Traumatic brain injury: Integrated approaches to improve prevention, clinical care, and research. Lancet Neurology, 16(12), 987–1048. 10.1016/S1474-4422(17)30371-X, 29122524

[bib62] Martin, E. M., Lu, W. C., Helmick, K., French, L., & Warden, D. L. (2008). Traumatic brain injuries sustained in the Afghanistan and Iraq wars. *Journal of Trauma Nursing*, 15(3), 94–99. 10.1097/01.JTN.0000337149.29549.28, 18820555

[bib63] Masri, S. (2020). *Roles of parvalbumin-expressing interneurons in physiological changes to primary auditory cortex after hearing loss and blast exposure* (Unpublished doctoral dissertation). The University of Arizona.

[bib64] May, H. G., Tsikonofilos, K., Donat, C. K., Sastre, M., Kozlov, A. S., Sharp, D. J., & Bruyns-Haylett, M. (2024). EEG hyperexcitability and hyperconnectivity linked to GABAergic inhibitory interneuron loss following traumatic brain injury. *Brain* Communications, 6(6), fcae385. 10.1093/braincomms/fcae385, 39605970 PMC11600960

[bib65] McFarlan, A. R., Chou, C. Y. C., Watanabe, A., Cherepacha, N., Haddad, M., Owens, H., & Sjöström, P. J. (2023). The plasticitome of cortical interneurons. Nature Reviews Neuroscience, 24(2), 80–97. 10.1038/s41583-022-00663-9, 36585520

[bib66] McGill, M., Hight, A. E., Watanabe, Y. L., Parthasarathy, A., Cai, D., Clayton, K., … Polley, D. B. (2022). Neural signatures of auditory hypersensitivity following acoustic trauma. Elife, 11, e80015. 10.7554/eLife.80015, 36111669 PMC9555866

[bib67] McGuire, J. L., DePasquale, E. A. K., Watanabe, M., Anwar, F., Ngwenya, L. B., Atluri, G., … Evanson, N. K. (2019). Chronic dysregulation of cortical and subcortical metabolism after experimental traumatic brain injury. Molecular Neurobiology, 56, 2908–2921. 10.1007/s12035-018-1276-5, 30069831 PMC7584385

[bib68] Mondino, A., González, J., Li, D., Mateos, D., Osorio, L., Cavelli, M., … Torterolo, P. (2024). Urethane anaesthesia exhibits neurophysiological correlates of unconsciousness and is distinct from sleep. European Journal of Neuroscience, 59(4), 483–501. 10.1111/ejn.15690, 35545450

[bib69] Muldoon, S. F., Bridgeford, E. W., & Bassett, D. S. (2016). Small-world propensity and weighted brain networks. Scientific Reports, 6(1), 22057. 10.1038/srep22057, 26912196 PMC4766852

[bib70] Mullen, T. (2012). Nitrc: Cleanline: Tool/resource info. Repérá à https://www.nitrc.org/projects/cleanline.

[bib71] Nguyen, T.-T. (2016). The characterisation of a shock tube system for blast injury studies (Unpublished doctoral dissertation). Imperial College London.

[bib72] Nguyen, T.-T., Pearce, A. P., Carpanen, D., Sory, D., Grigoriadis, G., Newell, N., … Masouros, S. D. (2019). Experimental platforms to study blast injury. Journal of the Royal Army Medical Corps, 165(1), 33–37. 10.1136/jramc-2018-000966, 29794172 PMC6581094

[bib74] Nguyen, T.-T. N., Sory, D. R., Amin, H. D., Rankin, S. M., & Proud, W. G. (2018). Platform development for primary blast injury studies. Trauma, 21(2), 141–146. 10.1177/1460408618776035

[bib73] Nguyen, T.-T. N., Wilgeroth, J. M., & Proud, W. G. (2014). Controlling blast wave generation in a shock tube for biological applications. In *Journal of physics: Conference series* (Vol. 500, p. 142025). 10.1088/1742-6596/500/14/142025

[bib75] Nichols, J., Bjorklund, G. R., Newbern, J., & Anderson, T. (2018). Parvalbumin fast-spiking interneurons are selectively altered by paediatric traumatic brain injury. Journal of Physiology, 596(7), 1277–1293. 10.1113/JP275393, 29333742 PMC5878227

[bib76] Onnela, J.-P., Saramäki, J., Kertész, J., & Kaski, K. (2005). Intensity and coherence of motifs in weighted complex networks. Physical Review E, 71(6), 065103. 10.1103/PhysRevE.71.065103, 16089800

[bib77] Paasonen, J., Stenroos, P., Salo, R. A., Kiviniemi, V., & Gröhn, O. (2018). Functional connectivity under six anesthesia protocols and the awake condition in rat brain. NeuroImage, 172, 9–20. 10.1016/j.neuroimage.2018.01.014, 29414498

[bib78] Pachitariu, M., Lyamzin, D. R., Sahani, M., & Lesica, N. A. (2015). State-dependent population coding in primary auditory cortex. Journal of Neuroscience, 35(5), 2058–2073. 10.1523/JNEUROSCI.3318-14.2015, 25653363 PMC4315834

[bib79] Pandit, A. S., Expert, P., Lambiotte, R., Bonnelle, V., Leech, R., Turkheimer, F. E., & Sharp, D. J. (2013). Traumatic brain injury impairs small-world topology. Neurology, 80(20), 1826–1833. 10.1212/WNL.0b013e3182929f38, 23596068 PMC3908350

[bib80] Pathak, A., Roy, D., & Banerjee, A. (2022). Whole-brain network models: From physics to bedside. Frontiers in Computational Neuroscience, 16, 866517. 10.3389/fncom.2022.866517, 35694610 PMC9180729

[bib81] Popelar, J., Grecova, J., Rybalko, N., & Syka, J. (2008). Comparison of noise-induced changes of auditory brainstem and middle latency response amplitudes in rats. Hearing Research, 245(1–2), 82–91. 10.1016/j.heares.2008.09.002, 18812219

[bib82] Reid, A. Y., Bragin, A., Giza, C. C., Staba, R. J., & Engel, J., Jr. (2016). The progression of electrophysiologic abnormalities during epileptogenesis after experimental traumatic brain injury. Epilepsia, 57(10), 1558–1567. 10.1111/epi.13486, 27495360 PMC5207033

[bib83] Rona, R. J., Jones, M., Fear, N. T., Hull, L., Murphy, D., Machell, L., … Wessely, S. (2012). Mild traumatic brain injury in UK military personnel returning from Afghanistan and Iraq: Cohort and cross-sectional analyses. Journal of Head Trauma Rehabilitation, 27(1), 33–44. 10.1097/HTR.0b013e318212f814, 22241066

[bib84] Roy, A., Bernier, R. A., Wang, J., Benson, M., French, J. J., Jr., Good, D. C., & Hillary, F. G. (2017). The evolution of cost-efficiency in neural networks during recovery from traumatic brain injury. PLoS One, 12(4), e0170541. 10.1371/journal.pone.0170541, 28422992 PMC5396850

[bib85] Rubinov, M., Kötter, R., Hagmann, P., & Sporns, O. (2009). Brain connectivity toolbox: A collection of complex network measurements and brain connectivity datasets. NeuroImage, 47, S169. 10.1016/S1053-8119(09)71822-1

[bib86] Rumschlag, J. A., & Razak, K. A. (2021). Age-related changes in event related potentials, steady state responses and temporal processing in the auditory cortex of mice with severe or mild hearing loss. Hearing Research, 412, 108380. 10.1016/j.heares.2021.108380, 34758398

[bib87] Sattin, R., Sasser, S., Sullivent, E., III, & Coronado, V. (2008). The epidemiology and triage of blast injuries. *Explosion and Blast-Related Injuries, Effects of Explosion and Blast from Military Operations and Acts of Terrorism*, 3–40.

[bib88] Schumacher, J. W., Schneider, D. M., & Woolley, S. M. N. (2011). Anesthetic state modulates excitability but not spectral tuning or neural discrimination in single auditory midbrain neurons. Journal of Neurophysiology, 106(2), 500–514. 10.1152/jn.01072.2010, 21543752 PMC3154814

[bib89] Sharp, D. J., Scott, G., & Leech, R. (2014). Network dysfunction after traumatic brain injury. Nature Reviews Neurology, 10(3), 156–166. 10.1038/nrneurol.2014.15, 24514870

[bib90] Shiga, T., Ikoma, K., Katoh, C., Isoyama, H., Matsuyama, T., Kuge, Y., … Tamaki, N. (2006). Loss of neuronal integrity: A cause of hypometabolism in patients with traumatic brain injury without mri abnormality in the chronic stage. European Journal of Nuclear Medicine and Molecular Imaging, 33(7), 817–822. 10.1007/s00259-005-0033-y, 16565846

[bib91] Silver, J. M., McAllister, T. W., & Arciniegas, D. B. (2009). Depression and cognitive complaints following mild traumatic brain injury. American Journal of Psychiatry, 166(6), 653–661. 10.1176/appi.ajp.2009.08111676, 19487401

[bib92] Smailovic, U., Koenig, T., Savitcheva, I., Chiotis, K., Nordberg, A., Blennow, K., … Jelic, V. (2020). Regional disconnection in Alzheimer dementia and amyloid-positive mild cognitive impairment: Association between EEG functional connectivity and brain glucose metabolism. Brain Connectivity, 10(10), 555–565. 10.1089/brain.2020.0785, 33073602 PMC7757561

[bib93] Sullivan, P. G., Keller, J. N., Mattson, M. P., & Scheff, S. W. (1998). Traumatic brain injury alters synaptic homeostasis: Implications for impaired mitochondrial and transport function. Journal of Neurotrauma, 15(10), 789–798. 10.1089/neu.1998.15.789, 9814635

[bib94] Tait, L., Lopes, M. A., Stothart, G., Baker, J., Kazanina, N., Zhang, J., & Goodfellow, M. (2021). A large-scale brain network mechanism for increased seizure propensity in Alzheimer’s disease. PLoS Computational Biology, 17(8), e1009252. 10.1371/journal.pcbi.1009252, 34379638 PMC8382184

[bib95] Toth, Z., Hollrigel, G. S., Gorcs, T., & Soltesz, I. (1997). Instantaneous perturbation of dentate interneuronal networks by a pressure wave-transient delivered to the neocortex. Journal of Neuroscience, 17(21), 8106–8117. 10.1523/JNEUROSCI.17-21-08106.1997, 9334386 PMC6573747

[bib96] Vallez Garcia, D., Otte, A., Dierckx, R. A. J. O., & Doorduin, J. (2016). Three month follow-up of rat mild traumatic brain injury: A combined [^18^f]FDG and [^11^c]PK11195 positron emission study. Journal of Neurotrauma, 33(20), 1855–1865. 10.1089/neu.2015.4230, 26756169

[bib97] van den Heuvel, M. P., Kahn, R. S., Goñi, J., & Sporns, O. (2012). High-cost, high-capacity backbone for global brain communication. Proceedings of the National Academy of Sciences, 109(28), 11372–11377. 10.1073/pnas.1203593109, 22711833 PMC3396547

[bib98] van den Heuvel, M. P., Sporns, O., Collin, G., Scheewe, T., Mandl, R. C. W., Cahn, W., … Kahn, R. S. (2013). Abnormal rich club organization and functional brain dynamics in schizophrenia. JAMA Psychiatry, 70(8), 783–792. 10.1001/jamapsychiatry.2013.1328, 23739835

[bib99] VanderWeele, T. J., & Mathur, M. B. (2019). Some desirable properties of the bonferroni correction: Is the bonferroni correction really so bad? American Journal of Epidemiology, 188(3), 617–618. 10.1093/aje/kwy250, 30452538 PMC6395159

[bib100] Váša, F., & Mišić, B. (2022). Null models in network neuroscience. Nature Reviews Neuroscience, 23(8), 493–504. 10.1038/s41583-022-00601-9, 35641793

[bib101] Vascak, M., Jin, X., Jacobs, K. M., & Povlishock, J. T. (2018). Mild traumatic brain injury induces structural and functional disconnection of local neocortical inhibitory networks via parvalbumin interneuron diffuse axonal injury. Cerebral Cortex, 28(5), 1625–1644. 10.1093/cercor/bhx058, 28334184 PMC5907353

[bib102] Verellen, R. M., & Cavazos, J. E. (2010). Post-traumatic epilepsy: An overview. Therapy, 7(5), 527–531. 10.2217/thy.10.57, 24761136 PMC3992621

[bib103] Verhelst, H., Vander Linden, C., De Pauw, T., Vingerhoets, G., & Caeyenberghs, K. (2018). Impaired rich club and increased local connectivity in children with traumatic brain injury: Local support for the rich? Human Brain Mapping, 39(7), 2800–2811. 10.1002/hbm.24041, 29528158 PMC6866640

[bib104] Vinck, M., Oostenveld, R., van Wingerden, M., Battaglia, F., & Pennartz, C. M. A. (2011). An improved index of phase-synchronization for electrophysiological data in the presence of volume-conduction, noise and sample-size bias. NeuroImage, 55(4), 1548–1565. 10.1016/j.neuroimage.2011.01.055, 21276857

[bib105] Wade, C., Ritenour, A., Eastridge, B., Young, L., Blackbone, L., & Holcomb, J. (2008). *Explosion injuries treated at combat support hospitals in the global war on terrorism. St Louis, MO: Explosion and blast-related injuries: Effects of explosion and blast from military operations and acts of terrorism*. Elsevier Inc.

[bib106] Wang, Y., Ma, L., Wang, X., & Qin, L. (2018). Differential modulation of the auditory steady state response and inhibitory gating by chloral hydrate anesthesia. Scientific Reports, 8(1), 3683. 10.1038/s41598-018-21920-x, 29487299 PMC5829141

[bib107] Watts, D. J., & Strogatz, S. H. (1998). Collective dynamics of ‘small-world’ networks. Nature, 393(6684), 440–442. 10.1038/30918, 9623998

[bib108] Xiong, Y., Gu, Q., Peterson, P. L., Muizelaar, J. P., & Lee, C. P. (1997). Mitochondrial dysfunction and calcium perturbation induced by traumatic brain injury. Journal of Neurotrauma, 14(1), 23–34. 10.1089/neu.1997.14.23, 9048308

[bib109] Zgorzalewicz, M. (2006). Long latency auditory evoked potentials in schoolchildren and adolescents with epilepsy. Przeglad Lekarski, 63, 8–13. 17471817

