# Supplementary material for: Alterations in topology, cost, and dynamics of gamma-band EEG functional networks in a preclinical model of traumatic brain injury
Source: Netw Neurosci. 2025 Jul 29;9(3):1013–38. doi: 10.1162/netn.a.21 (PMC12543302; doi:10.1162/netn.a.21)
Supplement: Supplementary file 1 [file netn-9-3-1013-s001.pdf]

Supplementary Materials

Subjects and Experimental Design

Subjects were male Sprague Dawley rats, sourced from Charles River Laboratories, UK. Upon arrival at the animal holding facility, they were maintained under standardized conditions with *ad libitum* access to rodent chow and sterilized water and a regular 12-hour light/12-hour dark cycle. There were allocated into 4 subgroups according to a 2-by-2 between subjects factorial design, with injury group (sham or blast) and post-injury timepoint (1 or 3 months post injury) as the factors. All procedures were conducted in accordance to the Animals (Scientific Procedures) Act under appropriate licenses, and in consultation with named veterinary surgeons, as well as named animal care and welfare officers.

Blast Injury

Blast injury was delivered using the shock tube located at the Centre for Blast Injury Studies at Imperial College London [11, 8, 9, 10, 5, 2, 4]. We have previously shown that this mild-to-moderate injury elicits hallmark electrophysiological and histopathological phenotypes [6]. Anesthesia was achieved via isoflurane and physiological parameters were monitored over a 20-minute period until stabilized, where stability was defined as the absence of pedal reflexes, a respiratory rate within the range of 50-60 breaths per minute, and an oxygen saturation exceeding 95%. While under continuous isoflurane delivery, rats were positioned on a steel platform attached to the open end of the compressed air-driven shock-tube. Orientation was lateral, with the right side directed towards the incoming shock-wave. Pressure waveforms captured using piezoelectric sensors positioned at the shock tube's exit, reflected a pattern consistent with the Friedlander model [12] with a peak overpressure of 232.58(6.45) kPa and positive phase duration of 1.39(0.04) ms (both values mean(sd)). Animals in the sham group underwent the same anesthetic protocol except for the blast exposure. Following the blast/sham procedure,

animals were recovered in a heated cage and given peri-operative buprenorphine analgesia for 72 hours (pre-blast: 0.05 mg/kg via subcutaneous injection, post-blast: *ad libitum* self-administration of 0.3 mg/kg buprenorphine jelly). Full details of the blasting and peri-operative procedures can be found in [6].

Electrophysiological Recordings

Terminal anaesthesia was induced using isoflurane, followed up by an introperitoneal injection of urethane (1.35 g/kg) for maintentance. Upon loss of the pedal reflex (usually within 1 hour), local anaesthetic was administered in the scalp area (bupicavaine 1.5 mg/kg) and atropine was administered subcutaneously (0.66 ml/kg, 1% w/v) to reduce mucous secretions. Subsequently, animals were transferred to a stereotaxic frame for surgery. A midline incision was made in the scalp using a scalpel, followed by blunt dissection of the connective tissues using scissors, clearing and smoothing of the dorsal surface of the skull with a blunt dental drill. All recordings were performed inside a shielded, anechoic chamber using Neuronexus electrodes (Rat EEG Functional), headstages (Neuronexus, SmartLink), and data acquisition system/software (SmartBox Pro/Radiens Allego). The EEG array was referenced to the nuchal musculature. Signals were bandpass filtered between 1.1 Hz and 15 kHz and sampled at 30 kHz.

Resting-State Data Preprocessing

Data preprocessing was performed using functions from the EEGLAB Toolbox [3] and custom scripts in Matlab (version 2019b). Raw data were imported into the workspace, converted into EEGLAB format and downsampled to 1 kHz (function *pop_resample*) to reduce computational load. Subsequently, line noise was suppressed through the *CleanLine* plugin [7, 1] (function *pop_cleanlinenoise*) applied twofold to the 50 Hz line frequency and its harmonics. The two applications were performed using two different moving window sizes, which was empirically found to be successful in eliminating line noise from the acquired data. Data were also subjected to automatic artifact rejection (function *clean_artifacts*). Data were then visually inspected to confirm the success of this automated step. As a final preprocessing step, data were decomposed using the Infomax ICA algorithm (function *pop_runica*) to seperate artifactual components. Components were visually inspected in terms of their temporal activation and spectra and com-

ponents containing primarily respiratory, cardiac or muscle-related artifacts were conservatively removed from the data. Datasets were excluded from further analysis in cases where line noise was not effectively suppressed, or the automated artifact rejection procedure led to more than 20% of the datapoints being rejected. 1 animal in the blast 1-month group and 2 animals in the sham 3-months group were excluded based on these criteria.

Phase-Based Functional Connectivity

Functional connectivity was assessed via the debiased weighted phase-lag index (dwPLI) [13], a frequency-resolved, phase-based metric that quantifies the consistency of phase differences between two time series in a time-resolved manner, using Matlab and Fieldtrip functions. The weighting of each phase difference by its magnitude in the computation of dwPLI serves to eliminate zero-phase connectivity, which might be attributed to volume conduction [13], making it suitable for sensor-level connectivity analysis. The calculation involved segmenting the signals into windows of 3 s duration and then computing the cross-spectral density for each electrode pair using Welch's method with a 1.5 s sliding window and 50% overlap (MATLAB function *cpsd*). These cross-spectra are subsequently used as input to the low-level the FieldTrip function *ft_connectivity_wpli*. Connectivity values for a frequency band are determined by averaging across the frequency bins specific to that band (gamma: 25-80 Hz).

Weight Gain

In order to assess physiological correlates of the normalized network cost metric, its association with weight gain was explored. In an effort to account for the effect of longer post-injury survival of the 3-month post injury group the weight gain (percent of weight at the time of blast gained by the time of the electrophysiology experiment) was divided by the number of months post-injury, yielding a weight gain per month measure.

Auditory Stimuli

Auditory stimuli were generated using Matlab at a sampling rate of 250 kHz and saved as *wav* files. Broadband clicks were created as rectangular pulses with a duration of 0.1 ms. Tone pips were generated as sinusoidal waves with a duration of 25 ms and a 5 ms cosine squared ramp. The tones

had frequencies of 10, 37.5, and 65 kHz at a fixed intensity. All stimuli had an interstimulus interval of 500 ms and were presented 1000 times with alternating polarities to ensure artifact-free data with sufficient signal-to-noise ratio. All stimuli were delivered through dynamic speakers (Avisoft, Vifa) interfacing with UltraSoundGate Player 116 (Avisoft) for playback and digital to analog conversion at 16 bits, and software (Avisoft, RECORDER USGH) running on a Windows computer. Speakers were positioned at a distance of 22 cm from the animal's ears. The intensity of the stimuli was calibrated to be 80 dB SPL at the ear level of the animal using an Avisoft CM24/CMPA condenser ultrasound microphone, previously calibrated using a sinusoidal 40 Hz reference signal generator (Avisoft). The synchronization of sound stimuli and electrophysiological recordings was achieved through analog TTL pulses sent from Player 116 to the data acquisition module (Neuronexus SmartBox Pro).

Auditory Stimulation Data Preprocessing

The preprocessing of data during auditory stimulation closely resembled that of resting-state data. Signals were downsampled to 2 kHz (function *pop_resample* with default settings, including an anti-aliasing filter at the new Nyquist frequency). Line noise was removed using the CleanLine plugin. An additional artifact rejection step involved identifying high-amplitude voltage excursions exceeding 400 μV, which were subsequently excluded. Subsequently, data were subjected to finite impulse response filtering (function *pop_eegfiltnew* with default parameters) between 3 and 300 Hz (cascade of low followed by high-pass Hamming windowed sinc filters, orders: 88 and 3300 respectively). Finally, data were segmented into epochs based on event markers (-10 to 490 ms, function *pop_epoch*) and the evoked potential waveforms extracted by taking the mean over trials.

Supplementary Figures

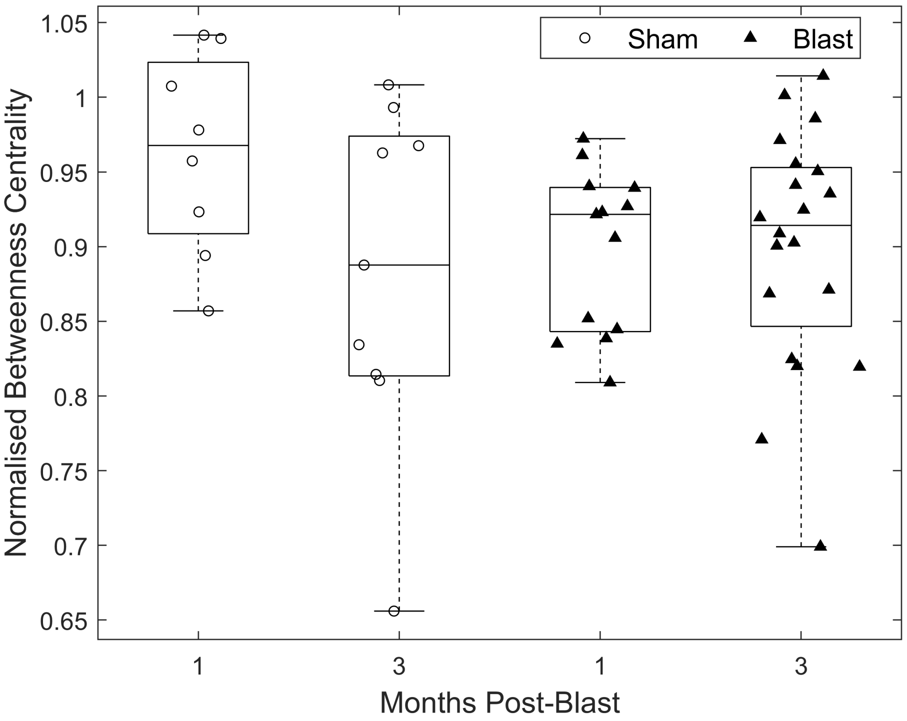

Figure 1: **No effect on normalized global betweenness centrality of gamma functional networks chronically after injury.** Permutation-based 2-way ANOVA (Group x Time Post-Blast with interaction, 10000 permutations).

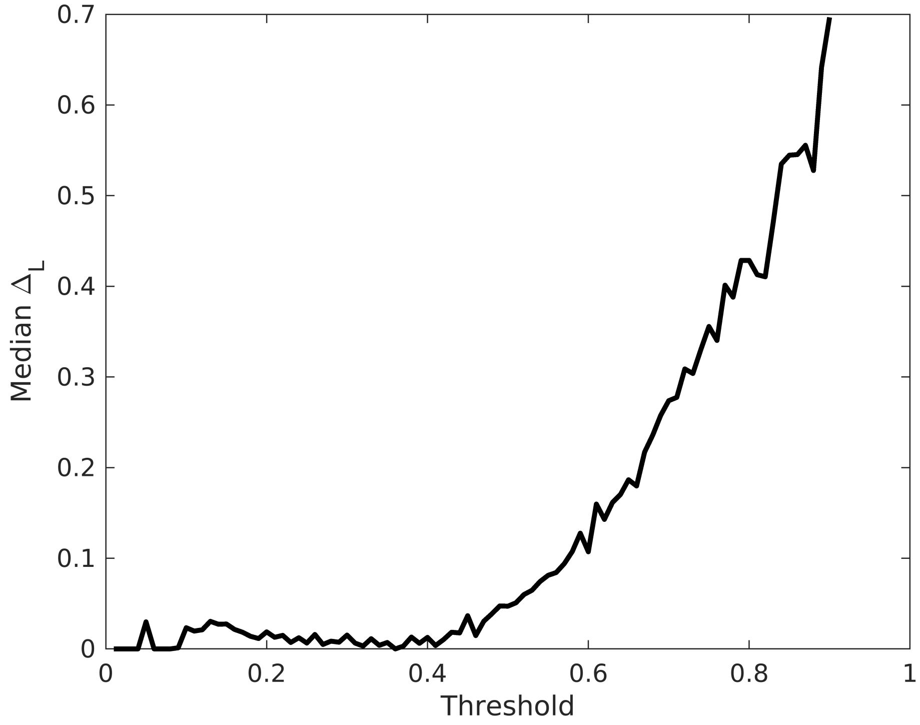

Figure 2: **Low fractional length of connectivity networks is robust to thresholding and binarizing.** We conducted a sensitivity analysis to assess the sensitivity of low fractional deviation from equivalent random networks (Figure 4C) to methodological choices. We thresholded our networks across a wide range of thresholds (0.01 to 0.9 in steps of 0.01), binarized them, and subsequently calculated fractional deviation from equivalent random networks as described in the main text (i.e., by randomly allocating the edges present). Across a wide range of thresholds, the mean fractional deviation, Δ_L, over our whole dataset remained low, as was observed from our main analysis on weighted, fully connected networks.

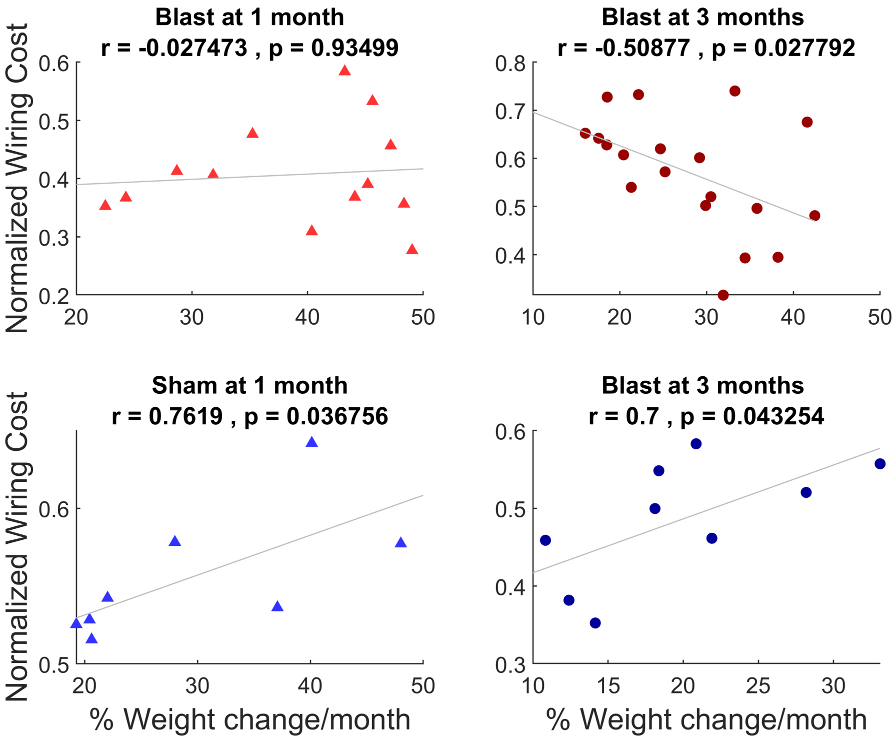

Figure 3: **Opposite correlation signs between normalized network cost and normalized weight gain between injury groups in the chronic phase is robust when considering all four subgroups. Top left:** blast at 1 month, **Top right:** blast at 3 months, **Bottom left:** sham at 1 month, **Bottom right:** sham at 3 months **All panels:** Spearman correlation values and associated p-values are reported. Lines are best fit lines using least-squares. (blast 1-month: n = 13, blast 3-month: n = 20, sham 1-month n= 8, sham 3 -month n = 9).

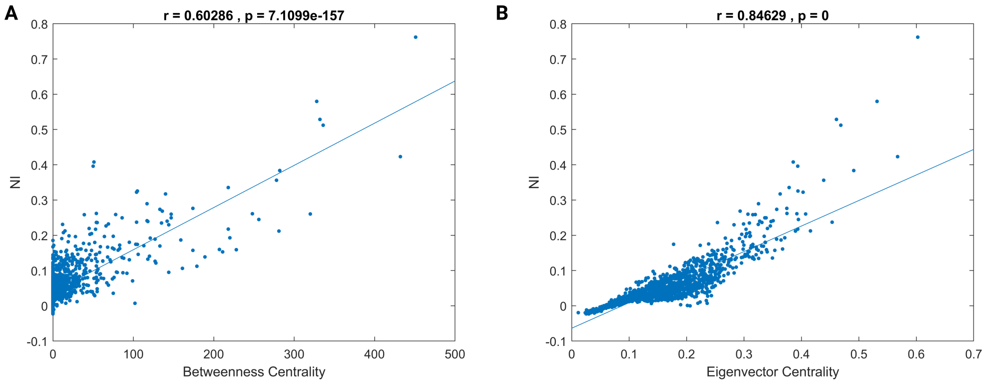

Figure 4: **Node ictogenicity correlates with centrality measures at the node level (A)** Betweenness centrality **(B)** Eigenvector centrality **All panels:** Spearman correlation values and associated p-values are reported.

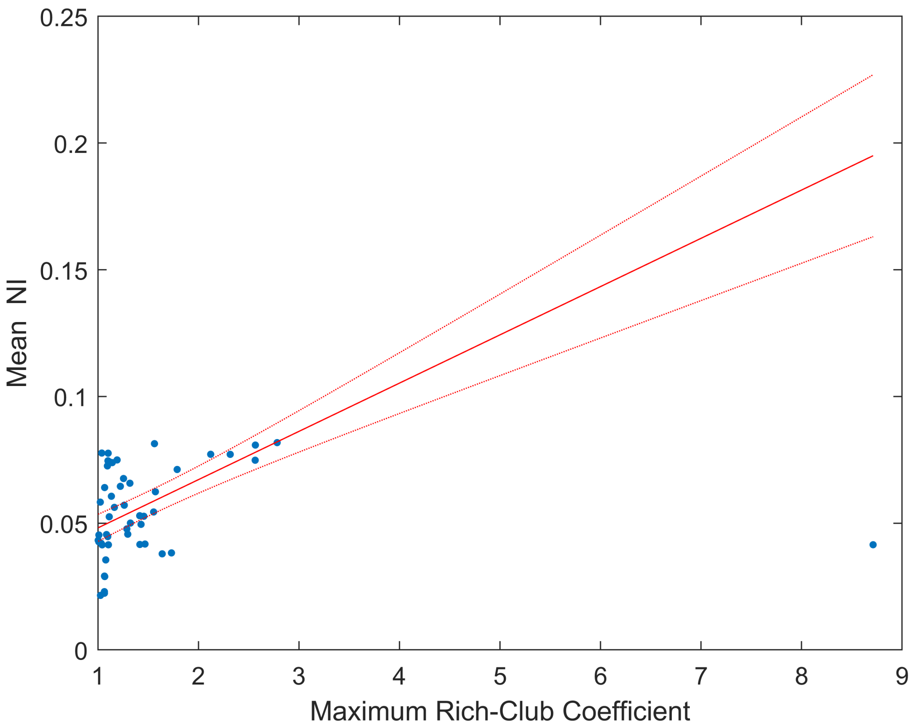

Figure 5: **Mean Node ictogenicity correlates with rich-club presence at the network/subject level.** Solid red line represents the line of best fit using robust regression, while dotted red lines represent the 95% confidence bounds.

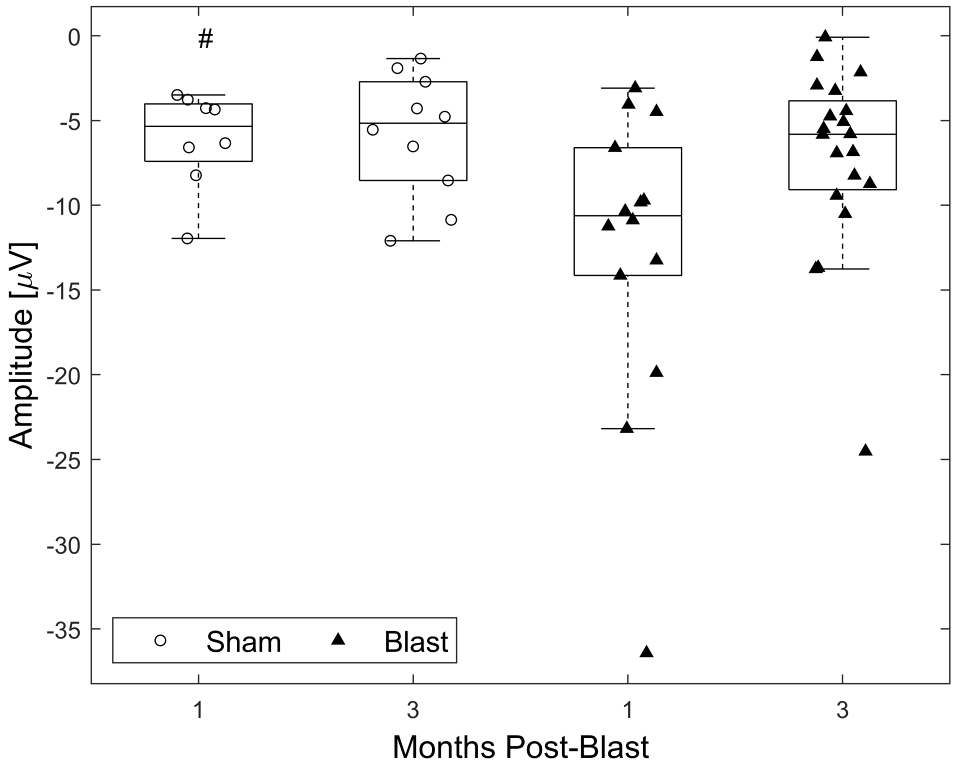

Figure 6: **Increased AEP N1 amplitudes following blast in response to 10 kHz tone pips.** Permutation-based 2-way ANOVA (Group x Time Post-Blast with interaction, 10000 permutations). Symbols denote p-values for a main effect of group. #: $p < 0.05$ (blast 1-month: n = 14, blast 3-month: n = 20, sham 1-month: n= 8, sham 3 -month: n = 10).

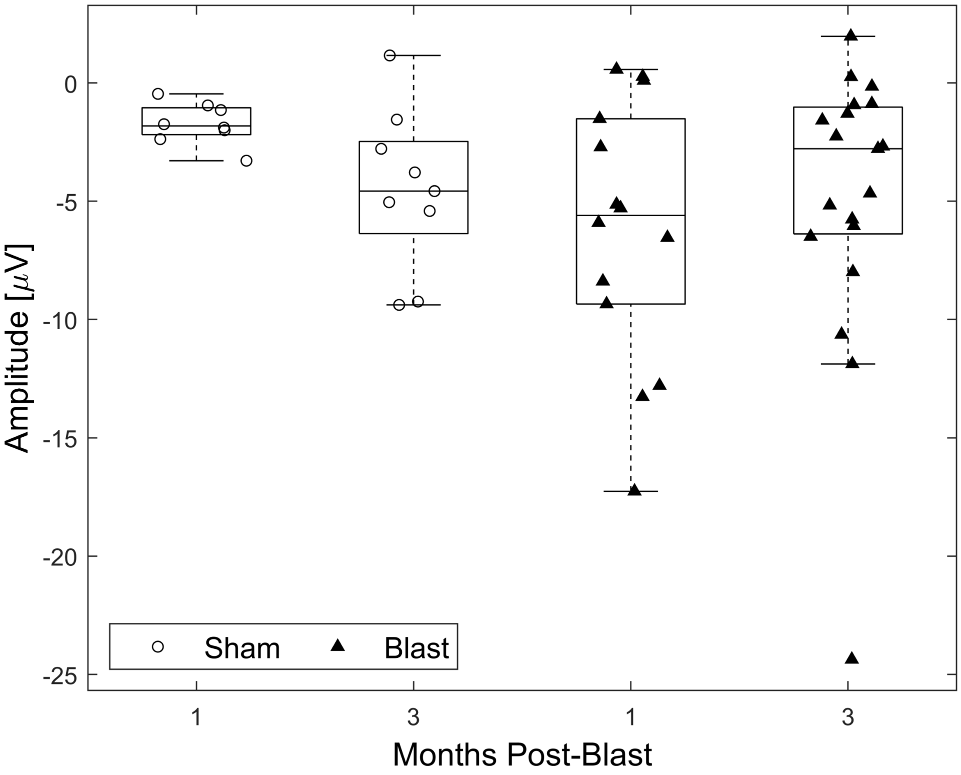

Figure 7: **No effect of blast on AEP N1 amplitude in response to 37.5 kHz tone pips.** Permutation-based 2-way ANOVA (Group x Time Post-Blast with interaction, 10000 permutations). (blast 1-month: n = 14, blast 3-month: n = 19, sham 1-month: n= 8, sham 3 -month: n = 9).

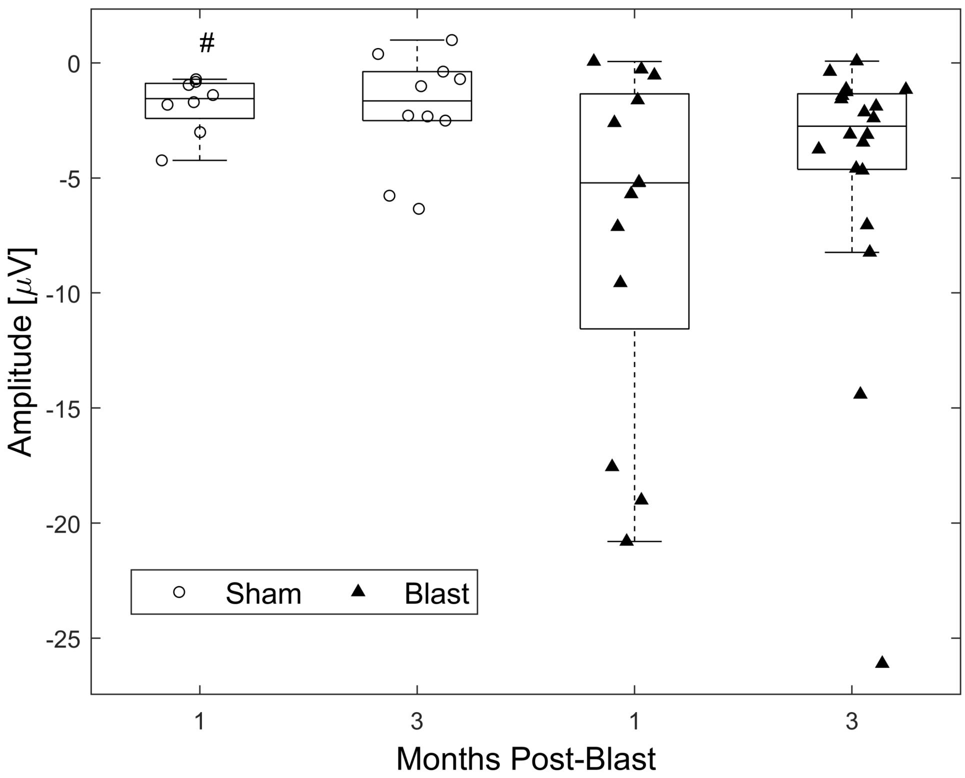

Figure 8: **Increased AEP N1 amplitudes following blast in response to 65 kHz tone pips.** Permutation-based 2-way ANOVA (Group x Time Post-Blast with interaction, 10000 permutations). Symbols denote p-values for a main effect of group. #: $p < 0.05$ (blast 1-month: n = 13, blast 3-month: n = 20, sham 1-month: n= 8, sham 3 -month: n = 10).

References

[1] Nima Bigdely-Shamlo et al. “The PREP pipeline: standardized preprocessing for large-scale EEG analysis”. In: *Frontiers in neuroinformatics* 9 (2015), p. 16.

[2] Rita Campos-Pires et al. “Repetitive, but not single, mild blast TBI causes persistent neurological impairments and selective cortical neuronal loss in rats”. In: *Brain sciences* 13.9 (2023), p. 1298.

[3] Arnaud Delorme and Scott Makeig. “EEGLAB: an open source toolbox for analysis of single-trial EEG dynamics including independent component analysis”. In: *Journal of neuroscience methods* 134.1 (2004), pp. 9–21.

[4] Theofano Eftaxiopoulou et al. “Prolonged but not short-duration blast waves elicit acute inflammation in a rodent model of primary blast limb trauma”. In: *Injury* 47.3 (2016), pp. 625–632.

[5] Zepur Kazezian et al. “Development of a rodent high-energy blast injury model for investigating conditions associated with traumatic amputations”. In: *Bone & Joint Research* 10.3 (2021), pp. 166–173.

[6] Hazel G May et al. “EEG hyperexcitability and hyperconnectivity linked to GABAergic inhibitory interneuron loss following traumatic brain injury”. In: *Brain Communications* 6.6 (2024), fcae385.

[7] T Mullen. “NITRC: cleanline: tool/resource info”. In: *Repéré à https://www. nitrc. org/projects/cleanline* (2012).

[8] Thuy-Tien Nguyen. “The characterisation of a shock tube system for blast injury studies”. PhD thesis. Imperial College London, 2016.

[9] Thuy-Tien Nguyen et al. “Experimental platforms to study blast injury”. In: *BMJ Military Health* 165.1 (2019), pp. 33–37.

[10] Thuy-Tien N Nguyen et al. “Platform development for primary blast injury studies”. In: *Trauma* 21.2 (2019), pp. 141–146.

[11] TT N Nguyen, JM Wilgeroth, and WG Proud. “Controlling blast wave generation in a shock tube for biological applications”. In: *Journal of Physics: Conference Series*. Vol. 500. 14. IOP Publishing. 2014, p. 142025.

[12] William G Proud. “The fundamentals of blast physics”. In: *Blast Injury Science and Engineering: A Guide for Clinicians and Researchers.* Springer, 2023, pp. 5–20.

[13] Martin Vinck et al. “An improved index of phase-synchronization for electrophysiological data in the presence of volume-conduction, noise and sample-size bias”. In: *Neuroimage* 55.4 (2011), pp. 1548–1565.
